# Predator-Prey Dynamics of Intra-Host Simian Immunodeficiency Virus Evolution Within the Untreated Host

**DOI:** 10.3389/fimmu.2021.709962

**Published:** 2021-10-06

**Authors:** Brittany Rife Magalis, Patrick Autissier, Kenneth C. Williams, Xinguang Chen, Cameron Browne, Marco Salemi

**Affiliations:** ^1^ Department of Pathology, Immunology, and Laboratory Medicine, University of Florida, Gainesville, FL, United States; ^2^ Emerging Pathogens Institute, University of Florida, Gainesville, FL, United States; ^3^ Department of Biology, Boston College, Chestnut Hill, MA, United States; ^4^ Department of Epidemiology, University of Florida, Gainesville, FL, United States; ^5^ Department of Mathematics, University of Louisiana at Lafayette, Lafayette, LA, United States

**Keywords:** HIV, rhesus macaque, untreated, intra-host, predator-prey, evolution, effective population size, phylodynamic

## Abstract

The dynamic nature of the SIV population during disease progression in the SIV/macaque model of AIDS and the factors responsible for its behavior have not been documented, largely owing to the lack of sufficient spatial and temporal sampling of both viral and host data from SIV-infected animals. In this study, we detail Bayesian coalescent inference of the changing collective intra-host viral effective population size (*N_e_*) from various tissues over the course of infection and its relationship with what we demonstrate is a continuously changing immune cell repertoire within the blood. Although the relative contribution of these factors varied among hosts and time points, the adaptive immune response best explained the overall periodic dynamic behavior of the effective virus population. Data exposing the nature of the relationship between the virus and immune cell populations revealed the plausibility of an eco-evolutionary mathematical model, which was able to mimic the large-scale oscillations in *N_e_* through virus escape from relatively few, early immunodominant responses, followed by slower escape from several subdominant and weakened immune populations. The results of this study suggest that SIV diversity within the untreated host is governed by a predator-prey relationship, wherein differing phases of infection are the result of adaptation in response to varying immune responses. Previous investigations into viral population dynamics using sequence data have focused on single estimates of the effective viral population size (*N_e_*) or point estimates over sparse sampling data to provide insight into the precise impact of immune selection on virus adaptive behavior. Herein, we describe the use of the coalescent phylogenetic frame- work to estimate the relative changes in *N_e_* over time in order to quantify the relationship with empirical data on the dynamic immune composition of the host. This relationship has allowed us to expand on earlier simulations to build a predator-prey model that explains the deterministic behavior of the virus over the course of disease progression. We show that sequential viral adaptation can occur in response to phases of varying immune pressure, providing a broader picture of the viral response throughout the entire course of progression to AIDS.

## Introduction

For RNA viruses such as human immunodeficiency virus (HIV) and its pathogenic simian relative (SIV), high mutation rates and short generation time are the constant fuel for rapid evolutionary change (1). The long-term fate of these changes, both among and within infected hosts (2), depends on the interplay of several population-level processes, such as genetic drift, selective forces from the environment, migration, and recombination (3). Evo- lutionary theory predicts that, for large populations, mutations occur frequently and their fate within the population is ultimately decided by the largely deterministic action(s) of natural selection. In contrast, in small populations, mutations are produced more rarely, with their fixation largely dependent on chance, stochastic events (genetic drift) (4). Determining which population genetic process (selection or drift) is the major driving force of viral population dynamics is critical to understanding the likelihood of immune escape and drug resistance in response to natural and synthetic antiviral defenses. Moreover, for viruses like HIV and SIV that persist for long periods of time, knowledge of this interplay is just as important during later stages of infection as it is at the time of transmission.

Experimental estimates of the height of the total HIV population size (*N)* within the host - 10^7^ 10^8^ HIV RNA-positive cells (5) - are consistent with a large population evolving in a deterministic fashion. However, one could imagine a few scenarios in which the truly effective size (*N_e_*) of the virus population is smaller than the total - i.e., not all productively infected cells produce virus that can reach a target cell in the vicinity; notably, a large fraction of the HIV genetic population has also shown to be defective, or incapable of replication, within the host owing to error-prone processes during viral replication inside the cell (6). *N_e_* can be estimated using a variety of population genetic approaches. For example, large changes in allele frequencies typically yield the smallest estimates of population size, and relatively small changes yield the largest population sizes (7). Similarly, the frequency of the nonrandom association of alleles at different loci tends to be higher in smaller population sizes. The study of allele frequency changes has led to relatively large estimates of *N_e_* (>10^4^) for HIV and thus support for deterministic influences on the replicating population (7, 8). Additional analysis of allele frequency changes over the course of 15 years showed clear patterns of major allelic shifts, or turnover, in the population, the relatively low rate of which (every 1, 000 days versus the short generation time of 1-2 days) is consistent with a large replicating population and deterministic processes (7). The rate of HIV population turnover using differing regions of the genome has also been estimated to be as frequent as 2.5 months (9) and to occur as early as acute infection (10).

An alternative, and perhaps more popular, approach to estimating *N_e_* using viral sequence data has been the time-varying coalescent model, which examines the temporal distribution of internal nodes, or coalescent events, within a reconstructed phylogeny dat- ing back to the time of the most recent common ancestral sequence (11). Genetic diversity provided from the sequence data and inferred coalescent tree can be used to estimate *N_e_* at intervals along the time-scaled phylogeny, including periods during which sampling is unattainable, by assuming that the estimated time to a coalescent event within the tree for two sequences is proportional to the population size. This approach has produced conflicting results regarding both *N_e_* estimates and thus the impact of selection on the population (7, 12–15). However, the seemingly paradoxical heavy influence of selection on a small population has been reconciled by da Silva (16) using a model of known HIV-specific parameters that affect population size (e.g., mutation rate, generation time), but also immune selection pressure targeting a large number of HIV epitopes (5) simultaneously. The HIV population present at initial infection (as low as 1 virion) is a result of the bottleneck associated with transmission (17). The evolution of this extremely small population might be expected to be influenced more by stochastic forces, limiting the diversity required to respond to environmental change. Yet, the virus is able to adapt quickly, expanding to peak viral load size within 21 28 days post-infection, owing likely to linked selection (18). In the absence of therapy, persistence of the virus, long after initial infection, eventually leads to immunosuppression through an evolutionary arms race between the pathogen and constantly stimulated immune cell populations (19). Estimating relative changes in viral *N_e_* over the full course of infection thus offer the potential to test relationship of outside factors and selection pressures on evolutionary and population dynamic (phylodynamic) trajectories (20, 21). The use of the coalescent framework applied to seasonal influenza (influenza A) to quantify population dynamics, for example, established that *N_e_* oscillates over time (in concert with regional outbreaks) as a result of the interplay between reassortment and periodic selective sweeps (22, 23). This oscillatory pattern is characterized by a ladder-like temporal distribution of taxa within the phylogeny, with temporal clusters separated by single, or few, lineages, representing extensive population turnover, such as was described by (7), as well as others (20, 24–27) for HIV and SIV (28) in the absence of therapy. The replacement of previous populations with the expansion of new lineages over the course of HIV/SIV infection suggests a continuation of highly adaptive behavior observed by (16), and similar to influenza A, in response to strong immune selective pressure. So while point estimates of genetic diversity from convenience sampling in HIV-infected individuals are often used to generate overall estimate of *N_e_* and assess the impact of selection, a more realistic scenario of progressive immune responses requires a time-varying viral population dynamic and may be best described using relative changes in *N_e_* over time.

Frequent sampling of the viral population increases the accuracy of phylodynamic inferences, as with any time-varying model, which can be provided with the use of an animal model of infection. However, the temporal structure is not the only potentially contributing factor to population dynamics. Given the systemic nature of HIV/SIV infection (29, 30), convenience sampling in HIV-infected individuals often limits longitudinal studies to the peripheral blood, neglecting infected tissues that can significantly contribute as a source of virus circulating in the bloodstream [e.g. (7, 31)], or act as a restrictive barrier of viral genetic exchange [reviewed in (3)]. Independent evolutionary processes in individual, highly restrictive tissues provide the opportunity for rapid changes in allele frequencies during lim- ited, short-term migration events (32). Given that individual tissues also harbor distinct immune cell compositions, viral population dynamics in the more commonly sampled blood need not accurately reflect the dynamic selective regime and evolutionary behavior of virus in remaining infected tissues (33). We, therefore, sought to explore SIV phylodynamics using viral RNA sequences sampled from a broader array of infected tissues at several time points over the course of disease progression in the macaque model of HIV infection. Using a much larger, more representative sample of the virus population within the host, we anticipated a larger estimate for the maximum effective population size than previously reported, with a dynamic pattern in *N_e_* over time distinct from that of the peripheral blood.

Though each tissue can harbor a distinct cellular repertoire, a highly systemic immune response and frequent exchange of virus among several largely infected tissues (34) would produce similar population turnover, and thus oscillating *N_e_*, in response to immune selection pressure. Population size oscillations in nature are often significant indicators of a deterministic predator-prey relationship, wherein two populations are in a continuous, alternating cycle of co-dependent growth and decline unless perturbed by an outside force (35, 36). Predator-prey dynamic models, where virus-infected cells are seen as prey and CD8+ T cells as predators, have been systematically investigated in the classic work of Nowak (37, 38). Moreover, it is well known that, in SIV infected macaques, transient artificial depletion of CD8+ T cells and natural killer cells (predatory sources of selection pressure) results in marked increase in viremia, which is again suppressed with the reappearance of SIV-specific CD8+ T cells (39). In a study involving tissues sampled from SIV-infected CD8+ T cells depleted macaques, near exponential growth of the SIV population (prey) prior to rapid progression to AIDS has also been described (40). Besides purely ecological predator-prey cycles, an oscillatory pattern in *N_e_* over time may also be expected in untreated HIV- and SIV-infected hosts as a response to the progressive series of immune responses (41–43), at least until the host immune system is nearly depleted and/or exhausted (44).

In what follows, we analyzed an extensive viral and immune dataset collected longitudinally from an immune-intact, SIV-infected macaque model of HIV infection over the entire course of disease progression. The relationship between SIV and primarily contributing immune population measures was then modeled to better understand the driving factor(s) in viral evolution and population dynamics in the absence of therapy.

## Results

Viral envelope *gp120* genetic sequences amplified from genomic RNA sampled over time from five distinct anatomical locations - plasma, bone marrow, lungs, and two isolated cell types within the blood (CD4+ T lymphocytes and monocytes) - were used to estimate the overall within-host, as well as tissue-specific, viral *N_e_* in each of eight macaques. Although we are aware that in HIV-infected patients the gp40 region does contain sites where mutations can lead to viral escape, we decided to focus on gp120 because, besides including known immunodominant epitopes as well as the CD4 binding domain, it also displays the highest phylogenetic signal, which is optimal for intra-host evolutionary studies in the SIV macaque model (45). Regression analyses and mathematical modeling were used to describe the relationships between Ne and the diverse repertoire of immune cell responses, represented by quantitative data on population size, over the course of disease progression.

### Incorporation of Sequences From Various Anatomical Locations Results in Highly Dynamic Total Intra-Host SIV Effective Population Size

**Figure 1:**
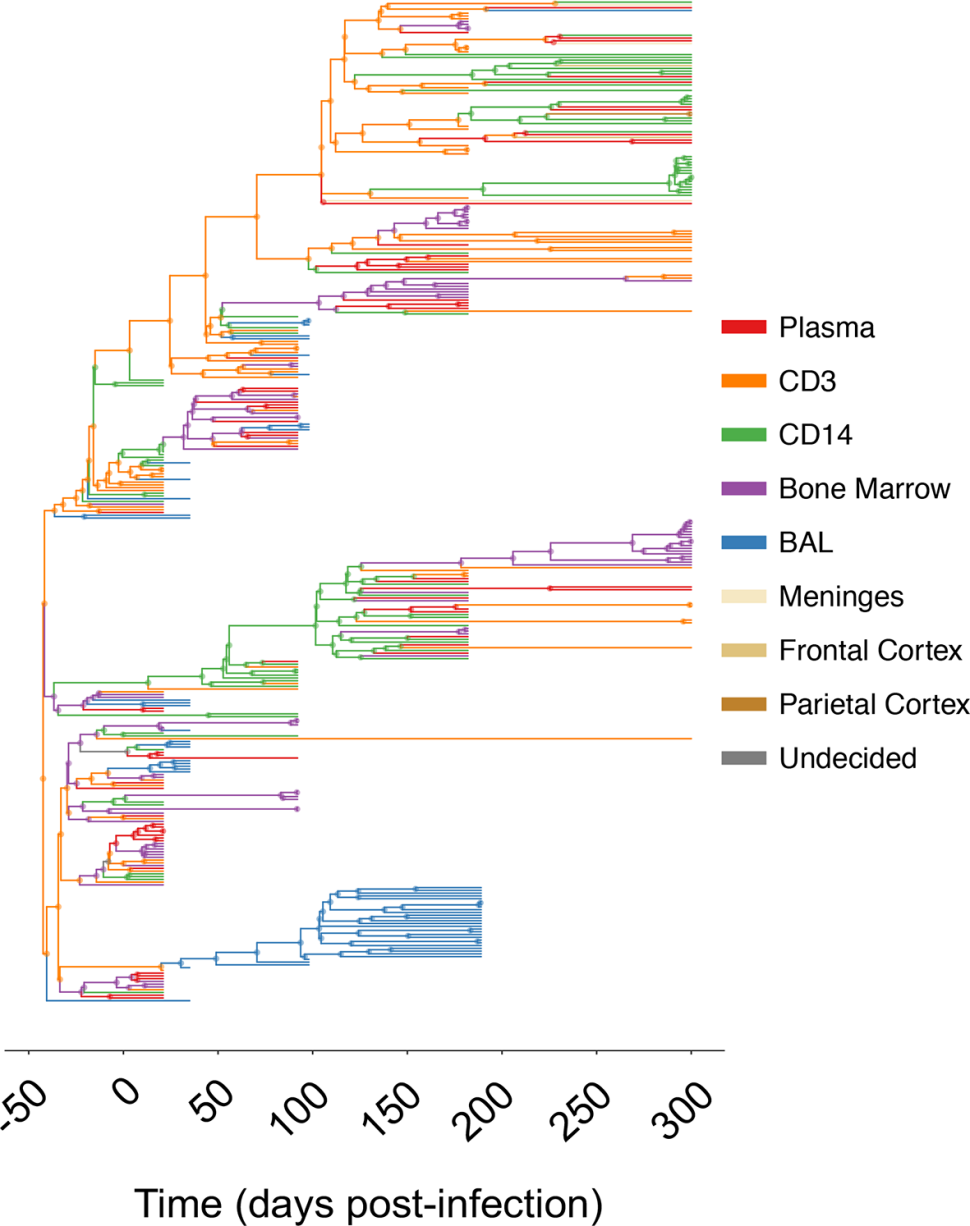
Maximum clade credibility (MCC) trees for combined and individual sampled tissue locations from SIV-infected macaque N09. The *gp120* MCC tree for macaque N09 was reconstructed using the Bayesian coalescent framework in BEAST v1.8 (47, 48) using all sampled tissues over time (x- axis). Branches are colored according to sampling origin (legend at right), with internal branches designated according to the highest posterior probability state using ancestral state reconstruction (2). MCC trees for remaining animals can be found in **Supplementary Material**.

**Figure 2:**
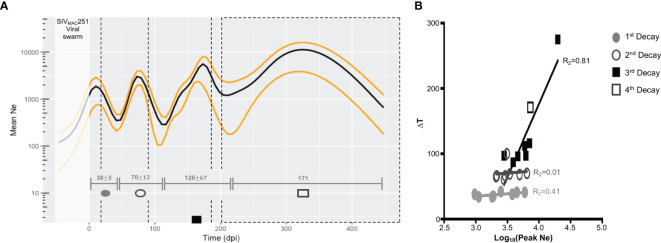
Changes in viral effective population size (*N_e_*) over the course of disease progression. **(A)** *N_e_* averaged over all macaques used in study. Median *N_e_* and high posterior density (HPD) intervals were inferred for each macaque *gp120* sequence alignment using the Bayesian coalescent framework in BEAST v1.8 (47, 48) for all sampled tissue locations over time (x-axis). *N_e_* (black), HPD (yellow), and time between lowest *N_e_* points (turnover time, grey intervals), were averaged across all macaques. Viral sampling times are represented by black, dashed vertical lines. represent 1 standard deviation from mean time interval. Time prior to infection (day 0 post-infection) represents the pre-transmission interval. **(B)** Relationship between turnover time and corresponding peak *N_e_*. The relationship of the length of the turnover time periods (∆T, y-axis) and log values of the peak *N_e_* (x-axis) within each turnover period was assessed using linear regression. As only one animal (N01) experienced a fourth turnover, corresponding values were included in the analysis of the third period. Each period is designated by a different shape and/or fill, and each point represents a single animal.

Viral sequence data from each anatomical location were combined for phylogenetic in- ference and estimation of the collective, or “total,” effective population size (*N_e_*) for each macaque. Although often 2-3 distinct lineages were observed for each macaque phylogeny, dating back as early as the pre-transmission interval (46) (**Figures 1** and **S1**), each of these lineages appeared to be temporally structured, giving rise to multiple population turnover events in the estimated within-host viral effective population size (*N_e_*) (**Figures 2A** and **S2**) and a periodicity demonstrated by auto-correlation (**Figure S3**). Peak *N_e_* values ranged from the previously observed estimate in the blood [10^3^ (7, 8, 13–15, 49)] to as high as 10^7^; however, collective *N_e_* values were consistently about one order of magnitude greater than that of plasma and/or PBMCs, supporting the hypothesis that a more representative sampling of the population was required to increase estimates of within-host *N_e_*.

Three to four distinct peaks in *N_e_* were observed, with mean time of peak *N_e_* across animals corresponding closely to sampling time (**Figure 2A**). When we looked for potential correlation between *N_e_* and sampling time, in each animal, an almost perfect, highly significant correlation was found for five out of eight animals (**Table S1**), while the remaining three had not significant correlation (p>0.05) with coefficients ranging between 0.07 and 0.87. Therefore, we cannot exclude that, at least in some animals, Ne estimates may be the result of a strong association between Ne and sampling times. Even more frequent sampling between existing time points is needed to determine if the number of peaks is underestimated. However, there is a trade-off between spatial and temporal sampling in animal models, as in- creased frequency of sampling across the tissues described in this study presents issues such as the confounding factor of immune stress resulting from slightly more invasive sampling (e.g., bone marrow) on disease progression, as well as cost. Given the similar mutation rate and generation times of HIV and SIV, as well as the average 3.3-month turnover time de- scribed in (9), we thought an increased sampling frequency strategy, relative to the current, an unnecessary risk. Furthermore, as described below, Ne estimates correlate in turn with oscillation in immune cell populations, which were measured independently through a much greater sampling density, strengthening our confidence in their biological meaningfulness.

We assume a significant impact of the existing immune pressures on the viral population, and thus, the observed sequential inflections in *N_e_* can be explained by the selective force(s) acting to reduce significantly 1) the size of the population through active cell killing or 2) the genetic diversity owing to fixation of adaptive allele(s), or both. Viral load measured in the blood, which typically acts as a proxy for the size of the total virus population, did not exhibit auto-correlation (**Figure S4**) indicative of time-scale periodicity, while the auto-correlation of Ne for all macaques was significant for a sequence of approximately equally spaced time lags. Therefore, we can infer that troughs in *N_e_* represent reduced genetic diversity in the population, and the time between each trough represents the population turnover time. For these animals, the mean intervals of turnover time increased with disease progression (38, 70, 126, and 171 days post-infection [dpi]) (**Figure 2A**). In contrast to the first two turnover time periods, characterized by virtually no variation in the length of the period between macaques, 81% of variation in length of the third and fourth periods could be explained by the peak *N_e_* estimate between population turnover (**Figure 2B**). This result suggests that the forces governing viral evolutionary dynamics during the early stage (100 days) of infection differ from those during the late stage, either qualitatively (e.g., innate versus adaptive immune response) or quantitatively (e.g., intensity of targeted immune selection), or both, for which additional immunological data and viral dynamic modeling were recruited.

### Periodic Change in SIV Intra-Host Effective Population Size Over Time Strongly Correlates With Immune Cell Population Dynamics

**Figure 3:**
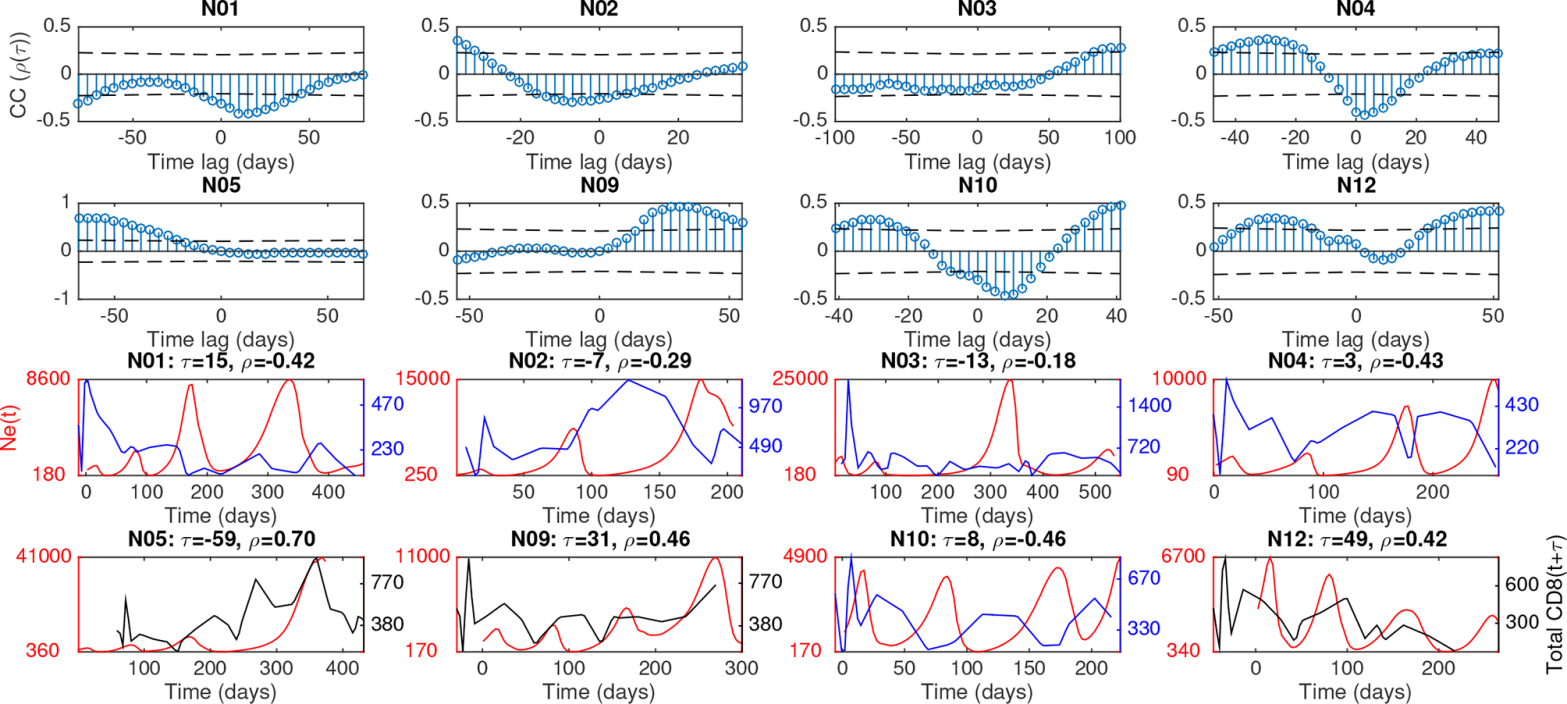
Cross-correlation of combined *N_e_* and total CD8 cells for each macaque, along with time-lagged plots at most significant phase-shift. Upper plots show correlation between *N_e_* (*t*) and *CD*8(*t* + *τ)*, where *τ* is time lag, along with dashed lines giving giving positive and negative correlation thresholds of significance (*p value* = 0.05). Lower plots show time-series, at the phase lag *τ* corresponding to most 550 days post-infection(dpi), the criteria for which included: significant correlation, of *N_e_* (*t*) (red) and *CD*8(*t* + *τ)* (black if positive correlation or blue if negative correlation).

As the decay of polymorphism can also occur purely through the process of genetic drift (4), we sought to more quantitatively define the relationship of *N_e_* with selection pressure through the longitudinal measurement of individual peripheral blood immune cell population sizes. Whereas viral sequences were obtained longitudinally at four-five time points during infection, blood samples containing immune population data could be taken easily at higher frequencies, ranging from 20-30 time points, depending on time of progression to SAIDS. Immune cells isolated from the peripheral blood at each of these time points were differentiated by markers specific to T cells, natural killer (NK) cells, monocytes, and B cells using fluorescence-activated cell sorting (see Methods). We allowed for a time lag between each pair of viral and immune data to account for natural phase shifts in reciprocal responses to respective population changes (presumably as a result of their prey-predator and evolutionary interactions) and adjust for uncertainty in estimates of the timing of *N_e_* inflections. The optimal time lag for each cell population was first chosen to maximize the correlation coefficient with *N_e_* in each animal (**Table S2**). Notably for most animals, the adaptive immune response populations (B and CD8+ cells) had significant periodic cross- correlation corresponding to their time-series lagging *behind* viral *N_e_* (denoted as negative time lag) by approximately 1/4 to 1/2 of the average period of oscillation (**Figures 3**, **S7** and **Table S2**).

**Figure 4:**
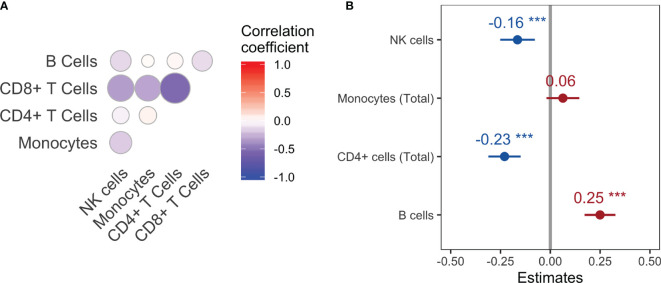
Correlation matrix **(A)** and correlation coefficient estimates **(B)** for each main cell population with viral *N_e_* using a linear random effects model. ***P-value<0.001.

In order to measure the strength of each cell population as a predictor of viral *N_e_* across macaques, consistency criteria for the optimal time lag were chosen so that the lag produced the same direction of correlation (positive or negative) and of lag (e.g., peak in *N_e_* consistently following that of the cell population) for that cell population across all animals. The direction of correlation and time lag were fixed for each cell type across macaques to maximize average correlation over all macaques. In other words, assuming similar mechanisms of response across all animals, extensive variation in the lag between animals would not be expected. Note that certain cell types, e.g. CD8+ cells, had reduced effects on viral Ne when employing the consistency condition due to differing sign of maximal correlation and corresponding time lag among all macaques, even though large cross-correlations were observed for several macaques. A random effects linear model was then used to quantify the strength of cell population data as predictors of viral *N_e_* (see Methods) over the entire span of disease progression across the macaque cohort. The correlation matrix from the effects model was first used to identify multicollinearity among the predictor variables (**Figure 4A**). A strong linear relationship between CD8+ T cells and the remaining cell populations, particularly CD4+ T cells (coefficient = 0.54), suggested an influence of this cell population on the variance of the remaining regression coefficients; CD8+ T cells were consequently removed from the model, revealing a statistically significant positive correlation of *N_e_* with the B cell population and negative correlation with NK cells and CD4+ T cells (**Figure 4B**). The strong positive correlation at negative time lags for B cells suggests again a co-evolutionary relationship with the virus, whereas the negative relationship for NK and CD4+ T cells is evidence of the known cell loss with increasing viral diversity that follows increasing viral replication. On the other hand, a statistically significant relationship with total monocytes was not observed.

As a growing body of evidence has supported distinct roles for sub-populations of major immune cell types (50), CD4+ and CD8+ T cells were further sorted into naive (N), central memory (CM), and effector memory (EM) cells, and monocytes into classical (CD14+CD16-), intermediate (CD14+CD16+), and non-classical (CD14-CD16+) sub-populations. Among CD8 sub-populations, EMCD8 exhibited the most significant cross- correlation (mean cc=0.48 and p-value=0.00322), whereas naive cells were slightly more cross-correlated with *N_e_* (mean cc=0.39) than other CD4+ sub-populations. Despite lack of significant correlation of the total monocyte cell population across all animals using the linear model, monocyte sub-populations were also analyzed (**Table S2**), as one or more contributing cell populations may be masked by an unrelated sub-population if the size of the latter is much larger. Indeed, correlation statistics for intermediate (mean cc=0.502, p=0.0015) and non-classical (mean cc=0.522, p=0.0022) monocytes (both CD16+) (**Figures S5, S6**), the minor sub-populations, were more significant than for classical (mean cc=0.324, p=0.104) monocytes (CD16-). A significant positive correlation was particularly observed for the fastest (300 dpi) progressing macaques, corroborating previous studies revealing a positive relationship between CD16+ monocyte population size with indicators of rapid disease progression - increased VL (51, 52) and reduced CD4+ T-cell count (51).

Although the relationship of immune cell population counts and *N_e_* in each animal appears strong, it is important to consider the contribution of exchange of virus between tissues and cell populations to the overall *N_e_*, as differing levels of exchange among a structured population can have significant effects on *N_e_* estimates (32). The number of transitions, or “jumps” between reconstructed tissue/cell origins within the Bayesian phylogenetic tree distribution (see Methods) was used as a proxy for migration, or exchange, events (53), revealing transient migration events, consistent with Feder et al. (2017) (33). A negative correlation (average correlation *ρ*=-0.304, p=0.0665) for each animal, with the exception of N02, was observed between *N_e_* and the number of jumps (**Figure S8**). Despite this relationship, significant overall meta-population structure within the maximum likelihood phylogeny was not observed, based on the inability to reject the hypothesis of a panmictic population when uncertainty in tree reconstruction was taken into account (refer to **Table S3** for macaques N01 and N03 and Rife Magalis et al. (2017) (28) for remaining macaques). In the absence of a purely structured population (i.e., no migration between any of five locations), lung macrophages and/or central nervous system (CNS)-derived virus were considered compartmentalized from remaining tissues in few macaques (28), suggesting that limited migration is still present and that these tissues may be driving the migration signal correlated with the population dynamics.

### A Two-Stage SIV Evolutionary Timeline Can Be Explained by Differing Immune Cell Population Dynamics

Because not every immune cell population is active at a given time during HIV infection (41–43), the strongest correlating cell population might be expected to differ during various disease stages. We next assessed the relationship of individual cell populations with viral *N_e_* during the two temporal stages described above (**Figure 2**) - the first stage corresponding to the first two invariable turnover periods, the second stage to the last two turnover periods, whose variability in length could be described by the magnitude of the corresponding peak *N_e_*. For each macaque, cross-correlation with cell populations was assessed during the first stage (**Table S4**) - the time interval ranging from infection to the second trough of viral *N_e_* (108 dpi), and the second stage (**Table S5**) - the remaining time until sacrifice. During the first temporal stage, NK cells and intermediate (CD14+CD16+) and non-classical (CD14-CD16+) monocyte populations were the strongest correlating cell types, signaling the influence of the innate immune response during early infection. In particular, the relationship between NK cells and *N_e_* exhibited an average correlation coefficient of *ρ* = 0.58 at optimal time lag (average *p* = 0.02), whereas intermediate and non-classical monocytes resulted in coefficients of *ρ* = 0.59 and *ρ* = 0.68, respectively (average *p* = 0.01 for both correlations). CD8+ T cells also strongly correlated with *N_e_* during the first stage at optimal time lag (*ρ* = 0.48, *p* = 0.03 on average), demonstrating the strength of the early CD8 response.

During the second stage, comprised of the third and fourth SIV population decay periods, B cells, CD4+ T cells, and CD8+ T cells were the strongest correlating cell types with viral *N_e_* (**Table S5**), signaling the influence of the adaptive immune response during chronic and end-stage infection. This finding is readily reconciled with increasing variance in time of peak *N_e_* across animals (**Figure 2**), possibly owing to host-specific differences in the virus specificity of the adaptive response. In particular, the relationship of B cells with SIV *N_e_* exhibited an average correlation coefficient *ρ* = 0.65 at optimal time lag (average *p* = 7 10*^−^* ^5^), suggesting a co-evolutionary relationship driven, at least in part, by the emergence of antibody escape mutants in the envelope gene, whereas CD4+ and CD8+ T cells resulted in coefficients of *ρ* = 0.7 and *ρ* = 0.66, respectively (average *p* = 1 × 10*^−^* ^4^ and *p* = 2 × 10*^−^* ^5^, respectively).

### Individual SIV-Infected Tissues and Cell Populations Are Characterized by Distinct Viral Population Dynamics

Virus sequenced in each sampled anatomical location were hypothesized to exhibit different population dynamic patterns from that of the total within-host population because these tissues and/or cells harbor smaller populations as compared to the entire host (and thus more of a role of genetic drift). Moreover, despite systemic regulation by CD8 cells and the potential for parallel evolution (54), each tissue may also be characterized by tissue-specific immune selection constraints. Consistent with these expectations, viral sub-population dynamics were highly tissue- and even macaque-specific (**Figure S2**). In the majority of macaques, plasma virus did exhibit significant periodicity, with peaks coinciding with that of total *N_e_* (mean cc=0.63 at mean lag = -9.7 days), the exception being macaques N05 and N10, suggesting plasma virus may be a reliable source of information as to the presence of population turnover and/or selection-driven events but not of the timing or strength of these events, as noted above. Though similar in timing to overall *N_e_*, plasma was not the only tissue location to exhibit an oscillating signal, suggesting other tissues contribute to the overall *N_e_* pattern and may experience similar, or systemic, selective pressures, as described above. However, the contributing tissue(s) was/were not the same across all macaques. For example, BAL *N_e_* was highly oscillatory in N12 (**Figure 2A**) but exhibited a pattern representative of logistic growth in N04. Macaque-specific tissue contributions may speak to inter-host differences in the localized balance of systemic and tissue-specific immune responses. Diving deeper, virus derived from sorted peripheral monocyte and T-cell populations can exhibit drasti- cally different population dynamics (**Figure S2B**), indicating differences in the evolutionary trajectory even within the same compartment (i.e., blood) (55–57). However, the comparative roles of genetic drift, local immune pressure, and infectivity have not been analyzed quantitatively for these smaller populations.

**Figure 5:**
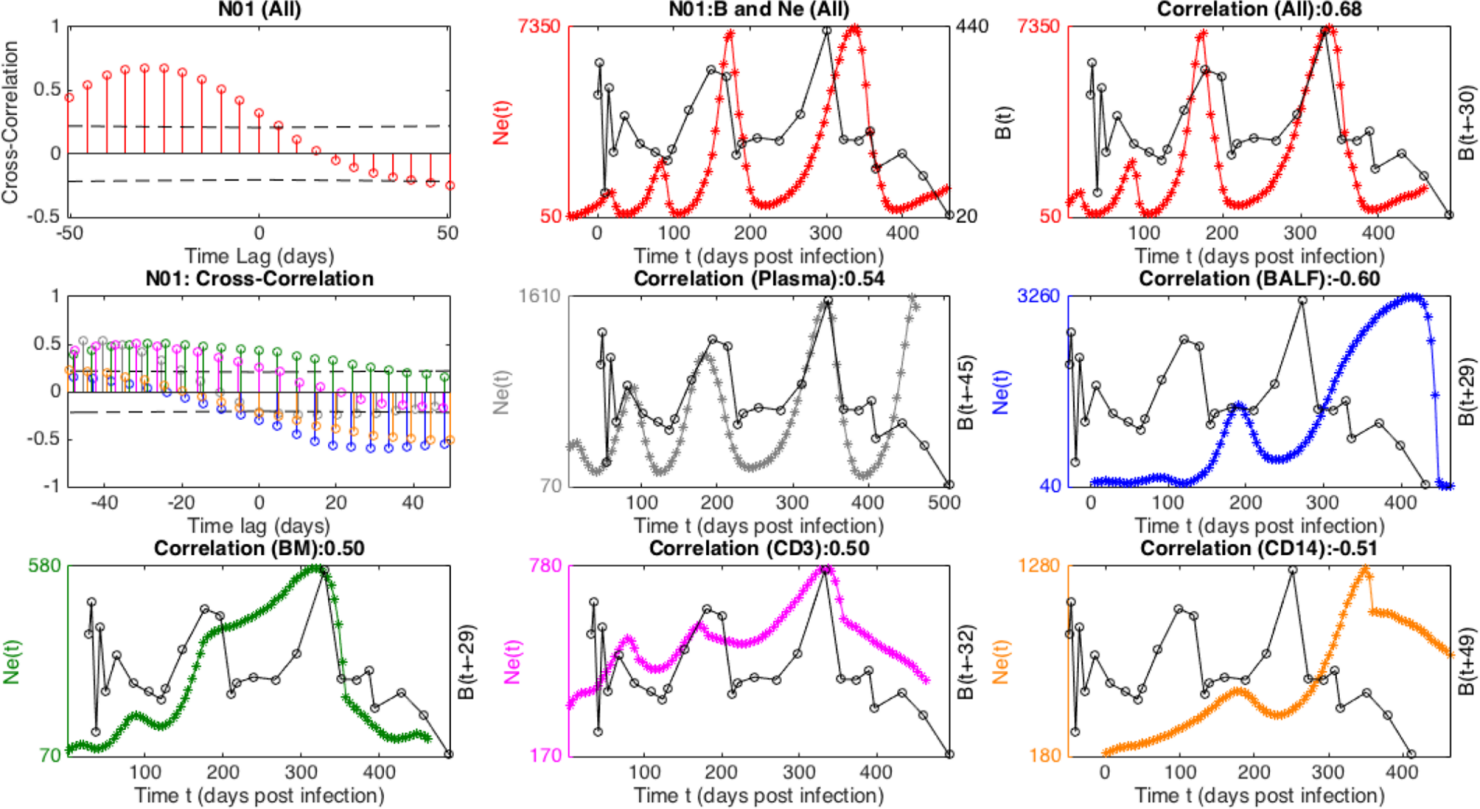
Cross-correlation of viral effective population size (*N_e_*), combined (All) and in different tissues, and B cell time-series for macaque N01. The top panel displays cross-correlation, and trajectories of combined *N_e_* with (unshifted and “optimally” time-lagged, respectively) B-cell population. The middle and lower panels contains analogous cross-correlations and time-lagged plots corresponding to distinct tissue *N_e_*, in particular Plasma (gray), BAL (blue), BM (green), CD3 (purple), CD14 (orange).

In general, *N_e_* derived from the individual tissues displayed less of an oscillatory signal than the total *N_e_*. Although individual sampled tissue and cell *N_e_* s exhibited strong cross- correlations with the immune cell population sizes, the power of inferring a relationship among the time series was diminished compared with the collective *N_e_* due to the relative decrease in large-scale periodicity. For example, in **Figure 5**, a striking time-lagged association between the viral *N_e_* and B-cell populations of N01 can be seen particularly when totaling over the sub-populations, indicative of continual prey-predator co-evolutionary interaction that may be expected under antibody driven selection. Overall, we performed wavelet cross-spectrum (WCS) analysis between *N_e_* from each individual sampling location and the immune cell time series (**Table S6**), a measure of the coherence of *N_e_* and the immune cell subsets at different timescales and stages of infection. The maximal amplitude of WCS was significantly larger for the total *N_e_* as compared to individual sampling locations, indicating a more robust periodic relationship for total *N_e_* and the immune cell counts.

### Eco-Evolutionary Model Simulations Recapitulate Cross-Correlated Oscillations in N_e_ and Immune Populations

We next considered a mathematical model for the eco-evolutionary dynamics of virus and immune populations in order to simulate the oscillations in viral *N_e_* and cross-correlations with distinct immune responses. Several previous works have modeled the evolution and in- teraction of multiple virus and immune response variants during HIV infection [e.g., Gusanov et al. (58) and van Deutekom et al. (59)]; however, simulations of large-scale fluctuations in viral diversity, underlying the dynamic *N_e_* observed in this study, have not yet been explored. The simulation model employed herein closely resembles that of da Silva (16), wherein estimates of immune-mediated cell death and patterns of fixation by viral escape mutants were used to simulate the effect of immune selection on *N_e_*, capturing the observed viral population dynamics during the early stage of infection. Similar to the consecutive fall and rise of *N_e_* observed in this study, the virus population during transmission from one host to another undergoes a drastic bottleneck (single transmitted genome) followed by rapid adaptation and subsequent expansion of the population during this early stage. We expand on this simulation model, describing a more dynamical system to account for oscillating patterns in viral and immune data. We build this model using a system of ordinary differential equations (see Methods), wherein viral variants are distinguished by a “binary sequence” of length *L* = *n* + *k* consisting of *n* epitopes each recognized by an (epitope-)specific immune response (taken to be CD8+ T-cells here), and *k* neutral loci not under selection pressure. The viruses infect a common target cell population (CD4+ T-cells) for replication and can undergo point mutations at each locus with some uniform probability rate. For neutralization of virus and clonal proliferation, the immune responses recognize their cognate epitope at (epitope-)specific mass-action rates, which also depend on the level of CD4+ T-cell help. A viral strain is either completely susceptible (0) or has evolved complete resistance (1) to immune recognition at a specific epitope, which incurs a fitness cost for the virus.

**Figure 6:**
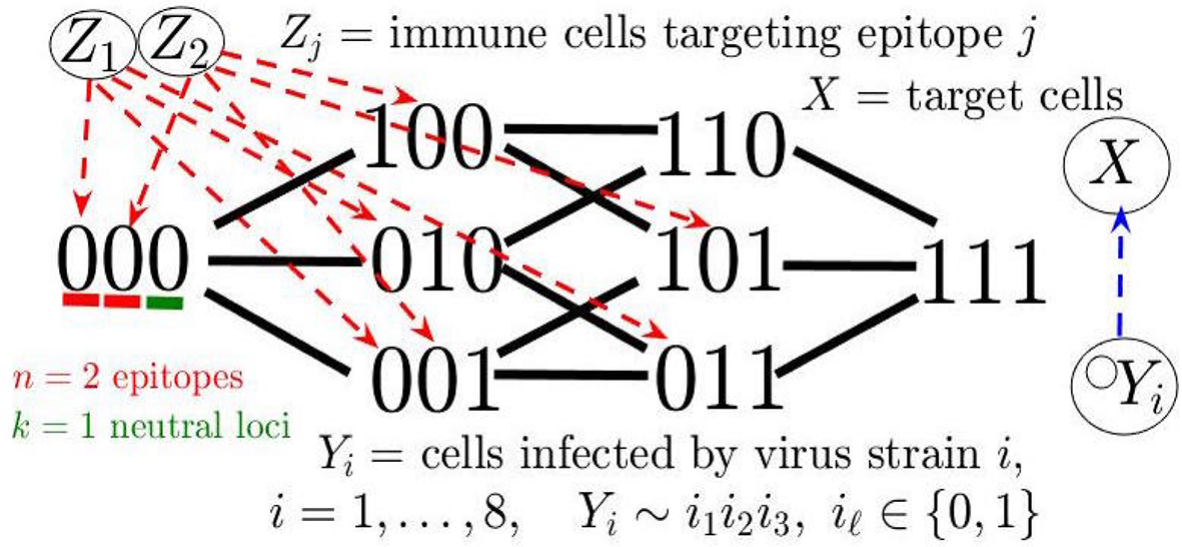
Diagram of mathematical model in the case of *n* = 2 epitopes and *k* = 1 neutral loci.

**Figure 7:**
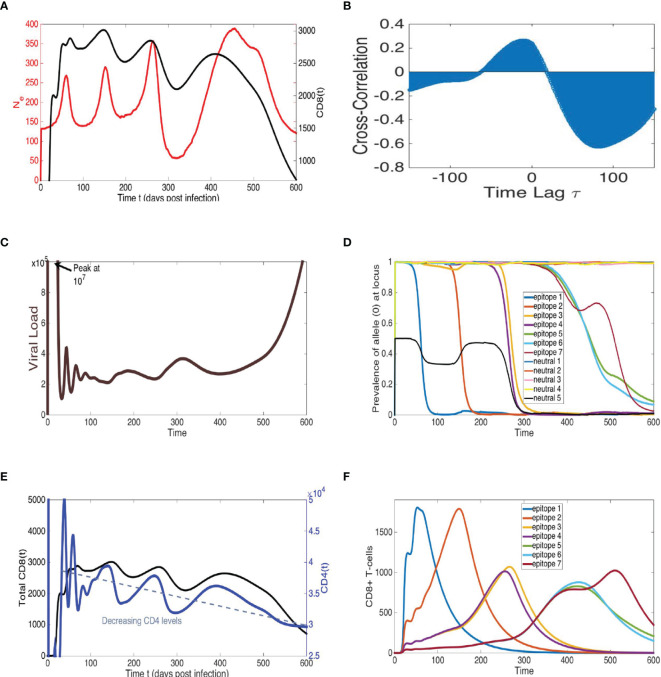
Model simulations recapitulate cross-correlated oscillations of *N_e_* and total CD8+ T-cells, sequential epitope escapes with viral diversity cycles of increasing amplitude, pre- cipitating a declining immune system and rapid viral growth at end-time, similar to AIDS progression. Representative simulation of multi-epitope virus-immune model (1) with viral mutation (see **Figure 6**) displaying trajectories of: **(A)** viral effective population size *N_e_*, calculated by proportionality rela- tionship with average loci genetic diversity (see Methods), and total CD8+ T-cells, **(B)** cross-correlation of *N_e_* and total CD8+ T-cells, **(C)** viral load (sum of all viral strain concentrations) and **(D)** allele distribution at each of *n* = 7 epitopes and *k* = 5 neutral loci, **(E)** total CD8+ T-cells, alongside target CD4+ T-cells, and **(F)** time-series of specific CD8 population for each epitope. The *n* = 7 epitopes are ordered according to an immunodominance heierarchy with identical immune strengths for epitopes *f* = 3, 4 and *f* = 5, 6, 7 with *r*_1_ = 1.05*, r*_2_ = 0.95*, r*_3_ = *r*_4_ = 0.84*, r*_5_ = *r*_6_ = *r*_7_ = 0.66 (10*^−^* ^4^). Clonal interference and accumulating viral diversity leads to more phase lag, larger period and amplitude in last two peaks of *N_e_* and total CD8+ T-cell time-series. Eventual escape from all epitopes coinciding with combined immune system decline leads to rapid viral growth at end-time. All other model parameter descriptions and values are listed in **Table S7**.

The model description so far yields our base model (see **Figure 6**), a special case of the general system consisting of *m* virus strains and *n* immune responses analyzed in (60). This model with simulated random mutation is sufficient for producing cross-correlated cycles in *Ne* and an adaptive (e.g., CD8) population (see Supplementary Information). In Addition, we extended the base model to recapitulate the oscillating decline in CD4 cells and rapid viral increase after immune escape (see **Figure 7**), characteristic of onset of AIDS and disease progression in some of the macaques in our study. In particular, our extended model accounts for a declining host immune system through pyroptosis, whereby non-permissive infected cells induce an inflammatory cascade, exacerbated by chronic immune presence, leading to excess CD4 cell death, which feeds back into the immune response by reducing an included term for CD4 help of immune response activation. Also, in accordance with the sampled immune cells in our experiments, we add a general innate immune compartment which targets all viral strains but is weaker than the adaptive response. Our detailed model description, along with additional simulations and discussion of alternative parameter/modeling choices, is contained in **Supplementary Information and Methods**.

The model depicts a heterogeneous viral population interacting with several immune responses that differ in strength determined by their immunodominance hierarchy, a key factor in driving viral evolution so that different combinations of multiple epitope escapes in the viral population rise and fall (60, 61). The model can, thus, be considered a hybrid of the Red Queen model (62), describing co-evolution of competing species, and the classical predator-prey model (35, 36), wherein these species are not competing but rather locked in a cycle of growth response based on species interaction. In order to measure *N_e_* in simulations, we utilize the relationship *π* = 2*N_e_E*, where *E* is the mutation probability rate per locus and *π* is genetic diversity averaged over all loci (see Methods). The qualitative pattern of sequential periods of large-scale oscillations in *N_e_* can be reproduced in the model, as illustrated with the example trajectory shown in **Figure 7**. The large amplitude fluctuations in *N_e_* reflect immune escapes as a dominant adaptive immune population (e.g., CD8) exerts pressure on the virus, driving diversification at this epitope until the resistant allele fixates, and the particular immune response decays. The process then repeats with the rise of a new population targeting a subdominant epitope. Viral load remains relatively constant during the chronic stage and does not match large-scale oscillatory behavior of *N_e_* (**Figure S9**), consistent with data. Furthermore, the CD4 T-cells steadily decline in concert with the total adaptive (CD8) immune population, until a weakened immune system no longer can control the prevalent multi-epitope resistant virus strains, and viral load rapidly grows in the last stage of infection.

An immunodominance hierarchy with successively weaker immune response, along with the overall diminishing CD4 help and increasing viral diversity, recapitulates increasing amplitude and period of oscillations in *N_e_*. Assuming measure of virus in the blood represents the overall behavior of the census population in the host, oscillations in *N_e_* may not necessarily reflect changes in census population size as much as they do patterns in viral diversification.If escape at a single locus is the predominant population genetic change occurring in the viral population, local peaks of the adaptive immune population and *N_e_* are generally aligned since the time of maximal diversity at the locus (reflecting 50% prevalence of epitope mutation) is coincidental with the maximum immune cell count targeting the epitope. Clonal interference can shift the *N_e_* peak since mutant fixation is delayed because of competition among viral strains along different lineages, as observed in **Figures 7B**, **S9**, **S10** and has been shown in other work [e.g. (63)]. The presence of concurrent exploration of several mutational pathways can explain larger phase lags of peak *N_e_* with respect to peak immune cell count observed in our data. Furthermore increased diversity and clonal interference may factor into the larger amplitude of *N_e_* as infection progresses. Indeed, there is some evidence in the data of larger phase lags between peak *N_e_* and CD8 during the second infection stage. Thus, our modeling suggests virus escape of relatively few early immunodominant responses, followed by slower escape of several subdominant and weakened immune populations with higher viral *N_e_* and competition among mutants. The model output is sensitive to parameters such as number of loci/epitopes and strength of immune responses, as also found in (16), and further explored in Supplementary Information and upcoming work. The complexity and host-dependence of several distinct immune responses, and viral epitopes and neutral loci, are likely to result in larger magnitude and variability in *N_e_*, and phase lags in cross-correlation.

## Discussion

As infrequent, and spatially restricted, sampling is available from human hosts, studies of HIV population and evolutionary dynamics are limited to one or few time points, often with a focus on the blood and/or the time prior to chronic infection (59, 61, 64). Viral evolutionary patterns over time and space, however, can be highly informative as to the size and structure of the replicating viral population, or effective population (*N_e_*), and how the virus is able to respond *via* adaptation to natural and synthetic antiviral defenses. Whereas previous studies in the context of HIV infection have relied on a single estimate of *N_e_* or limited point estimates across sparse sampling to distinguish between the deterministic or stochastic evolutionary forces at work, we turned to higher-resolution sampling in an animal model to estimate relative changes in *N_e_* over time. Importantly, these data have allowed for a phylodynamic study, incorporating empirical immunological data, of the contribution of host immunological factors in shaping viral population dynamics and disease progression. Previous studies, based on HIV sequences from peripheral blood of patients longitudinally sampled during early infection, have inferred larger intra-host viral *N_e_* (as high as 10^7^) values (7, 8) than the ones reported here for the SIV/macaque model. Such studies have provided support for the view of deterministic influences driving the virus population replicating within the host. Such a discrepancy, is not unexpected, as estimates of the true magnitude of *N_e_* are complicated in the presence of strong selection (8, 65). However, it is the reconstruction of *N_e_* highly periodic behavior, rather than its absolute magnitude, and its significant relationship with the similarly immune cell population dynamics that is crucial. In fact, the relationship of *N_e_* with immune data described herein can be considered an additional, strong line of evidence in support of deterministic viral behavior. Earlier work by da Silva (16) described similarly rapid adaptive behavior using estimates of *N_e_* during the first 100 days of infection, which was ascribed to an initially strong CD8 response to multiple (5) target epitopes simultaneously. da Silva was able to successfully capture the observed viral diversity with a waning CD8 response, defined by the rate of infected cell killing. The decline in the CD8 response is consistent with what we observed empirically using cell counts as a proxy for response, as well as with the simulation model parameters required to produce the *N_e_* behavior observed in this study. Expansion of the sequence data to the entire course of disease progression and inclusion of empirical immunological data revealed the continuing, periodic nature of the adaptive behavior observed by da Silva.

Though a strong correlation exists between the viral and cell population data, several intrinsic viral population variables offer an explanation for this highly dynamic *N_e_*, including variation in generation time or reproductive success; fluctuations in genetic composition; and/or variation in gene flow. Whereas gene flow, or migration, data estimated from the phylogeny suggest time-varying migration rates among a subset of tissues that potentially contribute to the estimation of *N_e_*, statistical analysis of the potential for spatial structure using the sequence data did not reject a panmictic, or highly mixed, population. The absence of significant overall compartmentalization of tissues but presence of time-varying transition rates among tissues in the phylogeny can be explained by both real and artefactual migration across tissues. In the latter scenario, shorter branch lengths due to strong selective pressure could result in a greater number of transitions between tissues along branches during the respective time intervals owing to the greater number of observable nodes. In the former scenario, selective sweeps imposed by immune cells capable of effectively acting systemically could lead to simultaneous subpopulation extinction followed by recolonization of adaptive strains (local bottleneck) (66). A subset of tissues (i.e., the similarly dynamic tissues) might undergo viral population extinction whilst others (the relatively stable tissues) promote migration of infected cells in response to immune selective pressure (relatively stable tissues). Indeed, similar timing in *N_e_* reduction was observed among more than one tissue coinciding with total *N_e_*, although the collection of contributing tissues varied according to time and individual subject. During the time of recolonization and a return to reduced migration rates, the temporary divergence of the subset of affected tissues would explain the reduced *N_e_* in the blood as compared with the collective *N_e_*. The temporary nature of this founder effect and small number of affected tissues could mask the compartmentalization signal during the test for panmixia among all sequences and tissues.

The simulation of genetic data with both time- and space-dependent rates of migration and episodic selective sweeps could aid in distinguishing true from artefactual gene flow. While structured coalescent models incorporating variation in gene flow rates exist (67, 68), sufficient statistical and computational power are needed to allow for variation across both space and time. Moreover, as these models are Bayesian in nature, prior probabilities describing *a priori* knowledge of migration rates involving each tissue is key. There exists limited empirical data as to anatomical migration patterns, even at the level of connectivity of circulatory systems and cellular movement involving these systems, although a recent study has quantified viral replication and migration rate in the brain (69). Once these data do become available, perhaps a coalescent model incorporating parallel evolution across compartments (i.e., owing to a systemic adaptive response) would not be far behind. As evidenced in this study, this model may be further complicated by varying sources of evolutionary constraint during differing stages of infection. The innate immune system (particularly NK cells and CD16+ monocytes) and the immunodominant adaptive CD8 and B cells appear to correlate with a rapid, yet predictable *N_e_* fluctuation during early infection. However, following this early stage, the time required to reduce SIV *N_e_*, potentially representing time to adaptation (66), is linked to, and may depend on, both the strength of the adaptive immune response and the level of viral genetic diversity accumulated at the time of this response.

Predator-prey interactions acting to drive changes in allele frequencies and sequential adaptive responses have been reported (70, 71) and are highly relevant to the study of intra- host HIV populations. Different from the classical paradigm of the predator oscillations being a quarter period *ahead* (positive time lag) of prey *population* cycles, the observed neg- ative time lag may reflect a predator-prey system with strong evolutionary and competitive features, as found with our mathematical model and also evidenced in other work examining impacts of clonal interference on immune escape (63, 72). Importantly, the strength of the CD8-virus signal also indicates a continued interaction over the full course of disease progression, characteristic of episodic viral adaptation to CD8+ and B cell responses specific to viral variants within each new rebounding population, even though no correlation has been found between CD8+ T cell response magnitude and escape rate (58). In particular, across all animals, the first peaks of *N_e_* and CD8+ cells line up, which can be explained by both peak viral load and escape of strong immunodominant response occurring around this time (10), whereas a consistent negative time lag appears between subsequent *N_e_* and CD8, matching our model simulations.

Our findings of large-scale temporal periodicity in intra-host SIV *N_e_* and connection with immune cell population activity thus provide a fresh perspective on HIV population dynamics, incorporating multiple predator-prey interactions driven by eco-evolutionary feedback between the virus and immune response. A note of caution, however, needs to be emphasized given the highly significant correlation we detected between *N_e_* estimates and sampling times in five of the eight animals included in the study. We cannot exclude that, at least in some animals, *N_e_* estimates may be an artifact of such a strong association. Nevertheless, *N_e_* estimates in each animal do correlate, in turn, with oscillation in immune cell populations, which were measured independently through a much greater sampling density, thus strengthening our confidence in their biological meaningfulness that reflects a predator-prey dynamic. Moreover, it is important to remember the limitations of current methods to estimate *N_e_*. Methods based on linkage disequilibrium or coalescent estimators that explicitly model selection can provide point estimates but are not capable of analyzing longitudinal data and would have missed *N_e_* dynamic behavior over time. On the other hand, the coalescent framework in BEAST that we used does capture *N_e_* oscillation dynamic but potentially underestimates the magnitude of *N_e_* peaks, which is an acceptable trad-off given the scope of the present work.

The variability described in this study is of particular interest for future work, as differing cross-correlation strengths and associated time lags point to host- and immune cell-specific factors that determine a network of interactive forces. Additional modeling can help to dissect these factors, for example, by incorporating distinct mechanisms involved in B- and T-cell responses, or inclusion of compartment-specific (as opposed to simply blood) immune concentrations. Analyses of the frequency and diversity of individual sites and their position relative to known CD8-targeted epitopes are also currently underway.

Though the results are readily explained by a predator-prey system, further studies on experimental inhibition of specific immune responses during the approximate turnover periods later in infection (**Figure 2A**) are necessary to test this hypothesis, particularly in the context of viral recombination and antiretroviral therapy (ARV). Recombination increases the genetic complexity, potentially accelerating adaptation and diversification of the viral population. ARV itself may be modeled as a new predator that alters the ability of the virus to reach *N_e_* levels sufficient for prolonged survival, and the emergence of novel viral populations in late-stage infection described in our study. Yet, knowledge of the time- dependent strength (and magnitude) of viral and immune responses in the absence of ARV may provide an opportunity for the development of a treatment strategy designed to evolve as would the natural, effective immune predator(s) with the end goal of prey extinction.

## Materials and Methods

### Study Population

Eight (N01-N05, N09, N10, N12) Indian rhesus macaques (Macaca mulatta) were infected intravenously with the viral swarm SIVmac251 (1 ng SIV p27) (73). All animals were euthanized at the onset of simian autoimmune deficiency syndrome (SAIDS) at ~200-550 days post-infection(dpi), the criteria for which included: 1) weight loss *>* 15% body weight in 2 weeks or *>* 30% body weight in 2 months, 2) documented opportunistic infection, 3) persistent anorexia *>* 3 days without explicable cause, 4) severe intractable diarrhea, progressive neurological signs, or significant cardiac and/or pulmonary signs, as previously described (74). Pathological diagnosis was determined post mortem by a veterinary pathologist. Diagnoses for N02, N04, N05, N09, N10, and N12 have been reported previously (75), whereas diagnoses for N01 and N03 can be found in **Table S8**.

### Ethical Guidelines

Animals were housed at the New England Primate Research Center, according to the standards of the American Association for Accreditation of Laboratory Animal Care and IACUC protocol #04802. Treatment of all animals was in accordance with the Guide for the Care and Use of Laboratory Animals of the Institute of Laboratory Animal Resources (76). Further detailed information on the handling and supervisional guidelines for the animal cohort can be found in Lamers et al. (2015) (45). All possible measures were taken to minimize discomfort of the animals, and the guidelines for humane euthanasia of rhesus macaques were followed.

### Sample Collection and Sequencing

Plasma and PBMCs, unelicited bronchoalveolar lavage fluid (BAL) macrophages, and bone marrow (BM) aspirates were collected at four time points - 21 dpi, 90 dpi, 180 dpi, and necropsy. Cryopreserved PBMCs were quickly thawed in a 37°C water bath before being transferred to a 50ml conical tube containing 40ml RPMI with 20% FBS pre-warmed at 37°C. Cells were washed twice and transferred to a FACS tube and stained for 15 minutes at room temperature with an antibody cocktail consisting of anti-CD14-Pacific Blue (clone M5E2), anti-CD3-Alexa Fluor 700 (clone SP34-2), anti-CD20-Cy7-APC (clone B27) and anti-CD16-Cy7-PE (clone 3G8) (all from BD Pharmingen, San Jose, CA), anti-HLA-DR- ECD (clone L243, Beckman Coulter, Miami, FL), and Live/Dead Aqua (Invitrogen, Eugene, OR). All antibodies were titrated to determine optimal concentrations. Antibody-capture beads (CompBeads, BD Biosciences) were used for single-color compensation controls for each reagent used in the study, with the exception of cells being used for anti-CD3 and Live/Dead Aqua. After staining, cells were washed once, filtered and resuspended in 1ml PBS. The BD FACSAria cytometer (BD Biosciences, San Jose, CA) was set up with a pressure of 20 psi and a 100-um nozzle was used. Instrument calibration was checked daily by use of rainbow fluorescent particles (BD Biosciences). After acquiring unstained and single-color control samples to calculate the compensation matrix, we acquired 1 x 106 events in order to set up the sorting gating strategy. CD14+ monocyte population were gated first based on FSC and SSC parameters, after which we excluded 1) dead cells by gating out Aqua+ cells and 2) unwanted cells by gating out CD3+ and CD20+ cells and then gated on HLA-DR+ cells. From the HLA-DR+ population, a dot plot of CD14 vs. CD16 was used to make a sorting gate, which included all monocytes except the CD14- CD16- subset. For CD3+ T-lymphocyte sorting, FSC and SSC parameters were used to gate lymphocytes, dead cells were excluded by using Aqua staining, and CD14+ cells were also excluded. Following this procedure, the CD3+ T-lymphocytes were gated based on CD3 expression and negativity for CD16. Post-sort purity were checked for each sample, and both CD14+ and CD3+ sorted subpopulations were *>* 98% pure. After cell sorting, the highly pure cell populations were washed with PBS twice and all liquid was aspirated. Cells were then stored as dry pellets at 80°C.

Viral genomic RNA was extracted from the longitudinal samples, as well as from the meninges and brain tissue sections at necropsy, as described previously (40, 45, 75, 77). Viral RNA envelope (*env)* glycoprotein *gp120* sequences were obtained using a modified single genome sequencing protocol based on previously published methods (78) for all samples. *env* was selected for its high phylogenetic informativeness (79), allowing us to perform phylodynamic inferences at the level of the host (28, 80), as well as for individual infected tissues and cell populations (**Table S9**). Furthermore, *env* is the primary target for the humoral host immune response, and is subject to extensive selective pressures (41), as the encoded protein is responsible for binding and entry into host cells and is thus highly exposed upon budding (21). Env *gp120* RNA sequences were aligned as previously described (40), and approximately 20 *gp120* sequences per tissue per time point were obtained after removal of potential recombinants. Detailed information regarding sample collection, sequencing protocols, and the sequence alignment procedure have been reported previously (75, 77). Sequence data for the majority of macaques and the inoculating viral swarm have been used for previous analyses (40, 45, 75) and all are accessible in GenBank (accession number designations are found in **Table S10**). Sequence alignments can be found in https://github.com/brmagalis/SIV_Phylodynamics/Population_Size/DATA.

### Bayesian Phylogenetic Inference

We note that the Bayesian phylodynamic analysis described herein was performed using the coalescent framework with relaxed molecular clock calibration and a number of simplifying assumptions, including the absence of significant gene-wide selection and meta-population structure, and a constant census viral population size. It is important to note that, while selection and varying census population size are not included as parametric constraints on the reconstruction of past population events within this framework, these phenomena can still be inferred indirectly, as they impact the evolutionary history and thus the contribution of past lineages to subsequent generations of virus, on which inference of effective population size is dependent. Since population structure can otherwise confound population dynamic inferences, we have incorporated downstream analysis of the relationship of effective population size with a common measure of the extent of population structure over time. Recombination is also a significant source of genetic diversity in an infected individual, and would be of interest in the estimation of viral *N_e_*. However, recombinant sequences cannot be resolved in a phylogenetic tree, posing significant problems for estimation of evolutionary parameters surrounding the recombination event. While network tree reconstruction tools exist for the inclusion of recombinant sequences (i.e., as a child of two parents instead of one), methods of reliable estimation of population dynamics using these network topologies do not yet exist. Therefore, sequences identified as putative recombinants [methodology described in Lamers et al. (81)] were removed from the alignments. Macaque-specific *gp120* sequence alignments for macaques N02-N05, N09, N10, and N12 were reported previously to contain sufficient phylogenetic and temporal resolution for Bayesian genealogical tree reconstruction with molecular clock calibration of internal tree nodes (28, 80). Similar results were observed for N01 and N03 macaque-specific alignments, which were not previously published, as well as individual tissues for each macaque (**Table S9**). Briefly, internal nodes of an initial maximum likelihood (ML) tree were determined to be well supported based on a maximum threshold of 10% unresolved quartet trees using likelihood mapping (82), and taxa showed evidence of increasing divergence from the most recent common ancestor of all sequences (sequenced inoculating viral swarm), as indicated by positive slope using linear regression analysis (83).

Bayesian tree reconstruction for each macaque alignment (including alignments for individual tissues within each macaque) was performed using BEAST v1.8.2 (47, 48) within the University of Florida’s high performance computing platform, assuming an uncorrelated re- laxed molecular clock model of evolutionary rate variation across branches (84) and Bayesian Skyride (non-parametric) demographic prior (85). Skyride is derived from the skyline-based family of coalescent Ne estimators, which act piece-wise to divide the time between the present and the root of each tree (within a distribution of likely trees) into segments during which the effective population size is defined as the inverse of the probability that each pair of lineages during that time share a common ancestor (i.e., coalesce), as described originally by Pybus et al. (11). Detailed prior information can be found in the representative xml also in GitHub. Effective Markov chain Monte Carlo (MCMC) sampling (14) for all Bayesian analyses was assessed by calculating the effective sample size (ESS) for each estimated parameter. ESS values *>* 200, calculated in Tracer (86), were considered suitable indicators of effective sampling. Estimated viral effective population sizes for each macaque can also be found in the github repository. Anatomical trait evolution was also inferred using an MCMC approach, and the number of transitions between discrete states, otherwise known as Markov jumps, between discrete states (i.e., cellular origin of sequence isolation) along branches of the tree were counted as previously described (53).

### Meta-Population Structure Analysis

The presence of tissue-specific meta-population structure was previously inferred for ML trees (rooted using SIVMAC239 reference sequence) belonging to macaques N02-N05, N09, N10, and N12 using two phylogeny-based methods - measures of tree correlation coefficient (TCC) and Simmonds Association Index (SAI) (28). Similar results were observed for N01 and N03 (**Table S3**).

### Time-Series Correlation Analysis

Cross-correlations of the distinct immune cell populations with the viral effective population size (*N_e_*) time-series were computed in each macaque. For each macaque, the genomic data sampled from distinct within-host sites was used to compute combined, or total, and distinct tissue viral *N_e_* time-series (utilizing BEAST software as described in previous section). The different immune cell populations were sampled (see above) at a distinct set of time points that are not equally spaced and less frequent compared to the time points at which *N_e_* is computed. In order to facilitate cross-correlation analysis between *N_e_* and the immune cell populations, the immune cell data was linearly interpolated onto the *N_e_* time points which were evenly spaced. The viral *N_e_* trajectories were smooth with some oscillatory signals. The extent of periodicity differed among tissues and macaques, but the oscillations tended to have large average period (time between consecutive peaks), with three to four definitive peaks for the total estimated *N_e_*. Autocorrelation of the total *N_e_* plots was performed to verify periodicity. The immune cell data are noisier than *N_e_*, however large period oscillations can still be visually detected in the time-series and their corresponding autocorrelation plots.

The cross-correlation of two stochastic processes *X* and *Y*, *ρ_XY_*, as a function of time lag, *τ*, is given by


$$\rho_{XY}(\tau )=\frac{E[(X_{t}-\mu_{X})(Y_{t+r}-\mu_{Y})]}{\left(\sigma_{X}\sigma_{Y}\right)},$$

where E denotes expected value (calculated as sample covariance above), *σ_X_*, *σ_Y_* are the sample variances, and *µ_X_*, *µ_Y_* are the sample variances, and µX,µY are the sample means. The “optimal” time lag of most significant correlation and corresponding time-shifted overlapping plots were investigated in order to detect relationship between viral *N_e_* and host immune population time-series datasets. Cross-correlation significance was assessed by a Student’s t-distribution based test, commonly utilized for Pearson’s correlation coefficient. Due to the inherent noise, we performed some pre-whitening (filtering) of the immune cell data and calculated the cross-correlations. However, the smoothness of the viral *N_e_* did not necessitate the pre-whitening, and calculation of optimal time lags appeared more visually accurate with the unfiltered data. Thus, the cross-correlation analysis with the unfiltered datasets was utilized. Additionally, we checked cross-correlation calculations with the viral *N_e_* data interpolated on to the immune cell data time points (containing less observation points). However this appeared less accurate in terms of the optimal time lag than the calculations with the other interpolation on to the more evenly spaced and more frequent viral *N_e_* time points.

A linear random effects model using the lme4 package in R (87) was used to evaluate the relationship of time-lagged cell population counts with viral *N_e_* across the cohort of macaques used in this study. Consistency criteria for the optimal time lag were chosen so that the lag produced the same direction of correlation (positive or negative) and of lag (e.g., peak in *N_e_* consistently following that of the cell population) for that cell population across all animals. Correlation directionality for each cell population was chosen based on maximizing average correlation over all macaques, with the optimal time lag chosen as the time at which the greatest significance in cross-correlation with *N_e_* was achieved.

We also performed wavelet spectral analysis to determine wavelet power and cross spectrum (WCS) and coherence of *N_e_* and the immune cell subsets at different timescales and stages of infection, as in Bigot et al. (88). We utilized complex Mortlet wavelet transform to smooth the time-series and computed the amplitude of WCS of the transformed time-series. The results largely concur with observations from cross-correlation and autocorrelation analysis for total *N_e_*, along with showing the particular intra-host time intervals and scales at which significant periodic relationships between different immune cell counts and *N_e_*. Additionally, the WCS amplitude for the individual tissue *N_e_* (with the distinct immune cell populations) was compared to total *N_e_*. **Table S6** contains values of this WCS amplitude for each *N_e_* and immune cell population, averaged across time in the different time-series and averaged across macaque for each immune populations. Additional wavelet spectral analysis output is available upon request.

### Mathematical Modeling

We consider the following extension of a general virus-immune dynamics model analyzed in Browne et al. (2018) (60), which includes a population of target cells (*X*), *m* competing virus strains (*Y_i_* denotes strain *i* infected cells), and *n* variants of adaptive immune response (*Z_j_*), along with an innate immune response (*W)*:


(1) $$\begin{array}{l} \frac{dX}{dt}=b-cX-X\sum_{i=1}^{m}\beta_{i}Y_{i}\left(1+h_{0}\left(W+\sum_{j=1}^{n}Z_{j}\right)\right),\\ \frac{dY_{i}}{dt}=\beta_{i}Y_{i}X-\delta_{i}Y_{i}-Y_{i}\sum_{j=1}^{n}r_{ij}Z_{j}-rY_{i}W,\;i=1,\ldots ,m\\ \frac{dZ_{j}}{dt}=q_{j}(1+c_{0}X)Z_{j}\sum_{i=1}^{m}r_{ij}Y_{i}-\mu_{j}Z_{j},\;j=1,\ldots n\\ \frac{dW}{dt}=qr(1+c_{0}X)W\sum_{i=1}^{m}Y_{i}-\mu W. \end{array}$$

Here we are assuming that virus load (the abundance of virions) is proportional to the amount of (productively) infected cells. This assumption has frequently been made for HIV since the dynamic of free virions occurs on a much faster time scale than the other variables. The function *f* (*X*) = *b-cX* represents the net growth rate of the uninfected cell popula- tion. The parameter *β_i_* is the infection rate and *δ_i_* is the decay rate for infected cells infected with virus strain *i*. The parameter *µ_j_* denotes the decay rate of the immune response pop- ulation *j*. We assume immune killing and activation rates are mass-action, representative of these events occurring as immune response cells recognize epitopes on the surface of in- fected cells. The parameter *r_ij_* describes the killing/interaction rate of immune population *Z_j_* on a strain-*i* infected cell, whereas *q_j_r_ij_* describes the corresponding activation rate for *Z_j_* (proportional to interaction rate *r_ij_*). Additional terms included in the model are the parameter *h*_0_ representing pyroptosis of CD4-cells dependent on chronic immune activation and cell infection, similar to Wang et al. (89), and *c*_0_ allowing for a factor of CD4 help in the immune response, and we explain the mechanisms motivating these additional terms involving *h*_0_ and *c*_0_ further below.

We suppose there are *m* = 2*^n^* ^+^ *^k^* possible viral mutant strains distinguished by infection rate *β_i_* and the binary string **i** 0, 1 *^n^* ^+^ *^k^* determining susceptibility (0) or resistance (1) against each of *n* epitopes or determining allele of *k* neutral loci. Inherent to the modeling framework is the viral fitness landscape, specifying the infection rate for each viral strain based on the fitness costs incurred for epitope resistance mutations encoded in the binary sequences. Similar to the methods in van Deutekom et al. (59), we simulate mutations of the *L* = *n* + *k* loci by drawing from a binomial distribution. We consider the following measure of genetic diversity. The probability *π* that two randomly sampled viruses differ in their allele at a particular locus, *P*, is given by *π *= 2*p *(1 *p_)_* where *p *is the frequency of the “0 allele” in the population at locus *P*. According to coalescent theory (90), averaging over a large number *L* of loci, $\pi =\frac{1}{L}\Sigma_{\ell =1}^{L}\pi_{\ell }$ ,gives the relationship *π* = 2*N_e_E*, where *E* is the mutation rate probability for each loci. With this relationship of effective population size, *N_e_*, and diversity, *π*, we compute *N_e_*, along with the cross-correlation with total immune response for each model simulation.

The number of epitopes simultaneously targeted by the immune system has been estimated from previous analyses of sequence data from all or most viral genes, which found escape mutations of up to five epitopes spreading to fixation simultaneously (91–93). Asquith et al. (94) and da Silva (16) have argued in favor of a potentially larger number owing to sampling-mediated underestimation. Due to computational limitations, we chose to consider *n* = 7 epitopes (with a maximum of 3 epitopes simultaneously fixating) and *k* = 5 neutral loci (**Figure 7**). The relatively small number of loci may tend to reduce *N_e_* compared with the actual observed data. We assume each epitope mutation imparts equal independent multiplicative fitness costs, i.e. if (*i*_1_… *i_n_*) represents the epitope sequence of strain *i*, then $\beta_{i}=\beta_{0}{\left(1-\kappa \right)}^{i_{1}+\cdots +i_{n}}$ , which corresponds to a positive epistasis that promotes a sequential nested immune escape trajectory (60). Furthermore in this example simulation, we prescribe a fixed immunodominance hierarchy with equal immune strength for epitopes 3 and 4, and epitopes 5,6,7, which allows us to illustrate the impact of clonal interference on phase lags between viral *Ne* and the immune population. The initial condition for the virus is set to be a mix of wild-type strains (all epitopes are susceptible), where the last neutral locus (locus *P* = *n* + *k* = 12) is varied so that 50% of its “1-allele” is in the initial viral swarm (see **Figure 7C**). All parameters and further model description, along with robustness check for cross-correlated oscillations of *Ne* and immune response under different fitness landscapes and parameter assumptions in the base model without pyroptosis, CD4 help or innate immunity [*h*_0_ = *c*_0_ = 0*, W* = 0 in system (1)]], are given in Supplementary Information.

To further explain our immunological assumptions, we first note that pyroptosis is the mechanism whereby non-permissive CD4 infection triggers the caspase-1 pathway, inducing pyroptosis, which can secrete inflammatory cytokines such as IL-1. These cytokines establish a chronic inflammation state and attract more CD4+ T cells to the inflamed sites, resulting in more infection and cell death. Thus, pyroptosis generates a vicious cycle in which dying CD4+ T cells secrete inflammatory signals that attract more CD4+ T cells to be infected and die. The process is exacerbated by chronic immune activation, hence inclusion of concentration of total immune response in the “pyroptosis factor” *h*_0_ for non-permissive CD4 infection rate. There is both experimental (=[Doitsh et al. (95)] and modeling [Wang et al. (89)] support for this mechanism driving AIDS progression. Furthermore, we note that although differentiating between non-specific and specific T-cells can make the model more complete, given that the model is already very complex, we choose to consider the overall CD4 population, along with (HIV-)specific CD8 cells. The (non-specific and specific) CD4 population (indirectly and directly) orchestrates overall immune response, hence the helper T-cell term *c*_0_ *X*. While Betts et al. (96), Wilson et al. (97), and Ogg et al. (98) report that 8-50% of CD8 cells are HIV-specific during acute infection, we did not evaluate viral specificity of the immune cells isolated from the peripheral blood in this study. Considering the dynamic nature of this immune cell population and correlation with viral *N_e_*, we model an adaptive immune population that emerges with differing specificity with each peak, or increase, in immune population size. However, we incorporate changes in only the strength of immune response in our model. Ogg et al. (98) have shown that the proportion of HIV- specific CD8 cells decreases with initiation antiretroviral therapy (ART). Hence, while we are unaware of changing proportions for the CD8+ and B cell population in the absence of ART, it is certainly possible that dynamic proportions over time can contribute to viral adaptation and should be considered in a similar model following the collection of data on temporal variation in these values.

## Data Availability Statement

Publicly available datasets were analyzed in this study. This data can be found here: Genbank accession numbers are provided in **Table S9**.

## Ethics Statement

The animal study was reviewed and approved by Institutional Animal Care and Use Committee, University of Florida.

## Author Contributions

Conceptualization, BR, CB, and MS. Methodology, BR, CB, and XC. Investigation, BR, CB, and PA.Writing – Original Draft, BR nad CB. Writing – Review and Editing, BR, CB, PA, KW, XC, and MS. Resources, MS and KW. Supervision, MS. Funding acquisition, BR, CB, and MS. All authors contributed to the article and approved the submitted version.

## Funding

This work was supported in part by the National Institute of Neurological Disease and Stroke at the National Institutes of Health (NIH) [R01 NS063897 to MS], the NIH National Institute of Mental Health [F31 MH109398 to BR], the National Science Foundation [DMS-1815095 to CB], and the Stephany W. Holloway University of Florida Chair in AIDS Research.

## Conflict of Interest

The authors declare that the research was conducted in the absence of any commercial or financial relationships that could be construed as a potential conflict of interest.

## Publisher’s Note

All claims expressed in this article are solely those of the authors and do not necessarily represent those of their affiliated organizations, or those of the publisher, the editors and the reviewers. Any product that may be evaluated in this article, or claim that may be made by its manufacturer, is not guaranteed or endorsed by the publisher.
