# Supplementary material for: Predator-Prey Dynamics of Intra-Host Simian Immunodeficiency Virus Evolution Within the Untreated Host
Source: Front Immunol. 2021 Oct 6;12:709962. doi: 10.3389/fimmu.2021.709962 (PMC8527182; doi:10.3389/fimmu.2021.709962)
Supplement: Supplementary file 1 [file DataSheet_1.pdf]

Supplementary Information

Additional Mathematical model details

The *basic reproduction number* of each virus strain in system (1) in main text can be defined as $\mathcal{R}_i = \frac{b\beta_i}{c\delta_i}$, but the overall fitness also depends upon immune resistance epitope mutations. For each virus strain Y_i, we associate a binary sequence of length $L = n + k$, $Y_i \sim \mathbf{i} = (i_1, i_2, \ldots, i_n, \ldots, i_L) \in \{0,1\}^L$, coding the allele type at each of n epitopes and L neutral loci. We assume that each immune response (Z_j) targets its specific epitope at the specific rate r_j for virus strains containing the wild-type (allele 0) epitope j, whereas Z_j completely loses ability to recognize strains with the mutant (allele 1) epitope j, i.e. for $Y_i \sim \mathbf{i} = (i_1, i_2, \ldots, i_n, \ldots, i_L)$:

$$r_{ij} = \begin{cases} r_j > 0 & \text{if} \quad i_j = 0 \\ 0 & \text{if} \quad i_j = 1 \end{cases}, \tag{1}$$

where $1 \leq i \leq m$ and $1 \leq j \leq n$. For example, a wild-type (founder) virus strain, denoted here by y_w, is represented by the sequence of all zeroes in the n epitopes since it is susceptible to attack by all immune responses. With assumption (1), we can define an *immune reproduction number* corresponding to each Z_j by $\mathcal{I}_j := r_j b q_j / \mu_j$. We say that immune response z_j is immunodominant over z_k if $\mathcal{I}_j > \mathcal{I}_k$ and assume without loss of generality the ordered *immunodominance hierarchy*: $\mathcal{I}_1 \geq \mathcal{I}_2 \geq \cdots \geq \mathcal{I}_n$.

Furthermore, there are $m = 2^L$ possible viral mutant strains distinguished by reproduction number $\mathcal{R}_i$ and the binary string $\mathbf{i} \in \{0,1\}^L$. The potential virus strains can be viewed in a mutational pathway network, mathematically an L-dimensional *hypercube graph*. Viral strains are connected by an edge if their sequences differ in exactly one loci, and thus are "one mutation" away from each other. An example model diagram depicting the viral strains in the hypercube graph, along with immune response populations targeting each epitope, is shown in Figure 6 of the main text. It is well known that mutation of an epitope comes with a viral fitness cost. In the particular simulation of Figure 7, we assume that each epitope mutation imparts equal independent multiplicative fitness costs; if virus strain Y_i has d epitope mutations (sequence $\mathbf{i}$ has d "1 alleles" in the n epitopes), then $\mathcal{R}_i = (1-\kappa)^d \mathcal{R}_0$ where $\mathcal{R}_0$ is the wild-type reproduction number and $0 < \kappa < 1$ is the fitness cost. We list the parameters utilized in this simulation in Table S6, which are representative of SIV/HIV infection.

We conducted further simulations for model (1), along with the base model where $h_0 = 0$ (no pyroptosis), $c_0 = 0$ (no CD4 help) and $W = 0$ (no innate immune response) in order to test robustness of model results under different parameter choices. In general, the full model (with $h_0, c_0 > 0$) is much more computationally costly than the base model. While both models generate large scale oscillations in Ne and total CD8 immune response, the full model additionally can yield characteristics of AIDS as mentioned in main text. By varying the number of epitopes, n, and specific immunodominance hierarchy, the number of peaks, the period and amplitude in N_e can change. First, we display in Figure S9 how changing the number of epitopes and immunodominance impacts the cross-correlated oscillations between N_e and CD8 cells in full model. In the case of $n = 4$ epitopes without any immune response of identical strength, we observe that although clonal interference does not delay viral immune

escapes, reversions among previously escaped epitopes can similarly create phase lags and increasing diversity. Both processes occurring at the same time can lead to increasing viral diversity N_e after a peak (and escape) of CD8 T-cells.

While the full model successfully mimics oscillations in N_e and can additionally produce hallmarks of AIDS progression, the pyroptosis and CD4 help terms are complex and the viral and immune fitness quantities are perhaps overly prescribed. In order to check for oscillations in more general scenarios, we also vary the fitness landscape in the base model by starting with uniformly distributed additive fitness costs and adding normally distributed pairwise interactions between epitopes, along with assuming uniformly distributed immune strengths $\mathcal{I}_j$. Furthermore, we vary the initial condition from no diversity in a neutral allele to having some neutral diversity, as we expect from the viral swarm used in our experiments, and additionally produce some simulations with compensatory mutations which allow virus to regain fitness cost from escape. Example simulations are displayed in Figure S10. Observe that oscillations in N_e and CD8 cells still occur in these simulations of the base model, reflecting viral resistance alleles being selected at multiple epitopes and subsequent partial reversions after dissipation of specific immune responses. Assuming compensatory mutations can drive more lasting epitope escapes (dependent on amount of fitness regained), and with or without compensatory mutations, the random fitness landscape and immunodominance hierarchy creates more irregularity in oscillations. Parameter values and assumptions of example simulations of both full model and base model in Figures S9 and S10 are provided in figure captions. Furthermore, several other simulations were conducted under various assumptions, and can be provided upon request.

The parameters used for the model are representative of SIV/HIV infection. However since this is a preliminary modeling effort attempting to illustrate a potential mechanism for the analyzed data, we do not perform thorough parameterization of the system and leave this for future work.

Note that while some parameter values are taken from literature, other parameters are calibrated to provide good qualitative fit to the data. Some parameter values are chosen for convenience. For instance, the number of epitopes and neutral loci, $L = n + k$, is chosen relatively small for faster computation, which has the effect of producing smaller values of N_e than observed in data. We might think that the total CD8 cells (or some type of adaptive immune response) targeting epitope j as possibly a cluster of epitopes considered together. Furthermore, infection rate, β_i, is tuned to match peak viral load on the order of 10^7.

The model was coded in MATLAB, where the built-in ODE solver ODE45 was utilized for simulations. With a mutation rate of $\epsilon = 1.67 \times 10^{-4}$ per site per day, we compute the number of mutations during replication as follows. We update mutations at fixed time steps, taken as $\Delta t = 1$ *day*, where we approximate the daily number of cells that become de novo infected per viral variant as $M_i = \beta_i X Y_i$ cells. To improve computation speed, we assume that only one of the L loci mutates per replication, i.e. the small probability of simultaneous mutations are neglected. Then for each viral variant $i = 1, \ldots, m$ and locus $\ell = 1, \ldots, L$, the number of mutations is given by $\mathrm{Bin}(M_i, \epsilon)$. The viral populations are updated accordingly, and the ODE solver is run for Δt time units and then the process repeats.

We briefly discuss our modeling choices over potential alternatives, although extensive

investigation is reserved for a future paper. First, note that the basic form of system (1) is based on the "standard virus model" which produces stable dynamics [12]. Different functional forms of target-cell dynamics, e.g. the logistic growth $f(X) = rX(1 - X/K)$, can induce population oscillations [14], however this is not supported by the relatively stable viral load observed. Another hypothesis is that sustained viral load oscillations were not detected by the sampling scheme, and ecological predator-prey cycles may drive characteristic cross-correlation phase lags in the data. However even models of eco-evolutionary dynamics, e.g. [6], do not produce the observed relatively large phase lags with immune response preceding N_e. In particular, although total prey (virus) size may be shifted to anti-phase cycles, the effective population size will oscillate in concert with the predator population, potentially with a small phase lag. Thus, we choose a model with fluctuations in viral diversity, and not in total viral load. Furthermore, the goal of computing N_e by method outlined in previous paragraph, along with a more realistic virus-immune interaction network, motivated our system over simpler multi-strain models, e.g. [11], without viral epitope sequences. Other variations in our model might also be considered, e.g. nonlinear immune activation functional forms as in [3], and we leave this for future work.

Table S1: **Linear regression of peak N_e times with sampling times for each macaque.**

Animal	R^2
N01	0.4311484
N02	0.07110985
N03	0.8730224
N04	0.9998805***
N05	0.9999319***
N09	0.9996271***
N10	0.9984341**
N12	0.9998011***

p-values: $<$ 0.001***, $<$0.01**, $<$0.05*

Table S2: **Most Significant Cross-correlations, P-values and time lag for Total N_e and distinct immune cell populations**

	B	NK	TotalCD4	NaiveCD4	CMCD4	EMCD4	TotalCD8	NaiveCD8	CMCD8	EMCD8	TotalMonos	CD14+CD16-	CD14+CD16+	CD14-CD16-
N01_cc	0.68	-0.46	-0.28	-0.31	-0.24	-0.19	-0.41	-0.28	-0.39	-0.5	-0.22	-0.25	0.17	0.34
N01_pval	8.28E-13	4.40E-05	8.62E-03	3.61E-03	2.88E-02	8.83E-02	7.67E-05	7.36E-03	1.95E-04	8.29E-07	4.04E-02	2.05E-02	1.16E-01	2.52E-03
N01_tlag	-30.32	90.96	30.32	30.32	30.32	65.7	15.16	10.11	15.16	15.16	35.37	35.37	-15.16	80.86
N02_cc	0.57	-0.44	-0.56	-0.45	-0.43	0.54	-0.28	-0.15	-0.61	-0.26	0.31	0.1	0.45	0.67
N02_pval	7.36E-09	2.03E-05	1.48E-08	1.12E-05	2.81E-05	2.04E-07	7.69E-03	1.57E-01	2.20E-10	1.69E-02	3.01E-03	3.65E-01	8.16E-06	1.08E-12
N02_tlag	-4.52	-9.04	-2.26	-6.78	0	18.07	-6.78	-9.04	0	-9.04	6.78	9.04	2.26	6.78
N03_cc	0.38	0.4	-0.32	-0.31	-0.26	-0.17	0.28	-0.27	0.24	0.45	-0.3	-0.29	0.31	0.29
N03_pval	3.81E-04	3.68E-04	6.57E-03	6.80E-03	2.36E-02	1.20E-01	1.95E-02	1.46E-02	4.90E-02	8.11E-05	6.07E-03	8.30E-03	9.88E-03	1.45E-02
N03_tlag	-31.28	81.32	-93.83	-93.83	-93.83	-25.02	100.08	-37.53	106.34	100.08	31.28	25.02	-112.59	-106.34
N04_cc	0.66	0.63	-0.26	-0.28	-0.19	-0.17	-0.37	-0.37	-0.34	0.61	0.39	-0.13	0.72	0.67
N04_pval	2.19E-11	3.67E-10	1.55E-02	7.74E-03	8.24E-02	1.33E-01	5.34E-04	4.14E-04	1.26E-03	2.15E-09	2.62E-04	2.60E-01	2.44E-14	7.97E-12
N04_tlag	-14.79	-20.71	0	0	0	8.88	2.96	0	0	-26.63	-11.84	29.59	-14.79	-14.79
N05_cc	0.67	-0.6	-0.36	-0.32	-0.25	0.35	0.69	0.55	0.61	0.73	-0.09	-0.32	0.67	0.58
N05_pval	6.63E-11	6.86E-10	6.00E-04	1.91E-03	1.68E-02	9.22E-04	4.17E-12	1.42E-07	9.66E-09	4.37E-13	3.94E-01	2.36E-03	3.83E-11	2.97E-08
N05_tlag	-58.53	0	0	0	0	0	-54.35	-45.99	-62.71	-75.26	-8.36	-8.36	-58.53	-54.35
N09_cc	0.49	0.46	-0.41	-0.47	-0.32	-0.18	0.39	-0.39	0.31	0.51	0.57	0.48	0.69	0.71
N09_pval	2.09E-06	1.31E-05	8.07E-05	4.74E-06	6.15E-03	1.30E-01	3.73E-04	2.17E-04	5.16E-03	1.06E-06	1.13E-08	3.92E-06	5.50E-12	1.75E-14
N09_tlag	3.43	20.6	0	0	-51.49	-54.92	30.89	0	20.6	24.03	6.87	6.87	-34.33	-6.87
N10_cc	-0.19	-0.35	-0.44	-0.49	-0.2	-0.2	-0.46	0.52	-0.37	-0.47	0.61	0.63	0.4	0.49
N10_pval	8.53E-02	1.22E-03	2.47E-05	2.08E-06	7.07E-02	7.18E-02	1.08E-05	3.45E-06	5.72E-04	7.56E-06	1.47E-08	5.55E-09	4.27E-04	9.78E-06
N10_tlag	0	2.57	0	2.57	0	0	7.71	41.1	-10.28	7.71	38.53	38.53	-35.96	-28.26
N12_cc	0.54	-0.64	0.46	0.47	0.47	0.76	0.42	0.65	0.24	0.31	0.46	0.39	-0.55	-0.35
N12_pval	5.24E-07	2.15E-10	2.08E-05	7.03E-05	1.25E-05	4.44E-16	4.01E-04	1.14E-09	4.44E-02	8.73E-03	4.47E-05	7.10E-04	1.04E-07	1.26E-03
N12_tlag	-16.21	6.48	-12.97	51.86	-12.97	-6.48	48.62	42.14	-29.17	-32.42	-32.42	-32.42	6.48	0
Avg_cc (abs. val)	0.52	0.5	0.39	0.39	0.29	0.32	0.41	0.4	0.39	0.48	0.37	0.32	0.5	0.51
Avg_pval	1.07E-02	2.08E-04	3.92E-03	2.52E-03	2.86E-02	6.81E-02	3.57E-03	2.24E-02	1.26E-02	3.22E-03	5.54E-02	8.21E-02	1.58E-02	2.28E-03
Avg_tlag (abs. val)	19.89	28.96	17.42	23.17	23.58	22.38	33.32	23.24	30.53	36.29	21.43	23.15	35.01	37.28

Table S3: **Compartmentalization of sequenced sampling locations for macaques N01 and N03**

Macaque	TCC[1] P(rb)	P(r)	SAI (BS)[2]
N01	**<0.01**	**<0.01**	0.58 (100)
N03	**<0.001**	**<0.001**	0.60 (100)

[1] Tree correlation coefficients (TCC) were calculated based on the number of branches (rb) or branch length (r) separating sequences within separate defined compartments. Statistical significance was determined using a null distribution of permutated sequences (1,000 permutations). P values of 0.01 were considered significant and are shown in bold.

[2] The Simmonds association index (SAI) represents the mean ratio for 100 bootstrap replicates of the association value calculated from the test sequences to that for 10 sample-reassigned controls. The association value (d) is defined as follows: d=(1-f)/2^{n-1}, where n is the number of sequences below the node and f is the frequency of the most common sample type. The bootstrap support (BS) for SAI values is also provided. SIVmac239 was used as a reference sequence for SAI determination. BS values of 80% (highlighted in bold) were considered significant. NA, not available.

Table S4: **Most significant p-values for total N_e and distinct immune cell populations during stage 1**

Cell Population	N01	N02	N03	N04	N05	N09	N10	N12	Average p-val
B	0.12645644	0.078833039	0.0392936	0.13216886	0.113147815	0.123356253	0.120089339	0.000128685	0.091684254
NK	0.11911638	2.98E-08	0.001833768	0.014065381	0.030836426	0.002921151	0.000171469	1.67E-05	0.02112016
Total CD4	0.290768964	0.072204838	0.009593187	0.238165719	0.060033812	0.15062199	0.016574205	0.00231081	0.105034191
Naive CD4	0.234389479	0.000571766	0.011980841	0.179266682	0.031711716	0.09170131	0.006542339	0.000813198	0.069622166
CM CD4	0.285486019	0.004198288	0.010763614	0.159703586	0.059608609	0.024822159	0.007758221	0.007849294	0.070023724
EM CD4	0.092083251	4.73E-13	0.000144466	0.013021352	0.040844657	0.022563094	0.049848687	2.80E-08	0.027313192
Total CD8	0.018634004	5.08E-09	0.022893532	0.004309257	0.101794936	0.02186737	0.038251138	0.025735413	0.029185707
Naive CD8	0.126589557	1.30E-08	0.054718529	0.075885036	0.001362672	0.015257865	0.047930026	5.74E-07	0.040218034
CM CD8	0.011462988	1.74E-07	0.061727615	0.001777004	0.012732097	0.209306825	0.038862577	0.258530866	0.074300018
EM CD8	0.007840874	2.76E-08	0.006065202	0.001428262	0.085670986	0.006735296	0.001231453	0.182802994	0.036471887
Total Monos	0.019228202	2.41E-09	0.084306409	0.01094742	0.002893546	0.029918452	0.037190563	0.004826577	0.023663897
CD14+CD16-	0.04296905	7.84E-09	0.152135655	0.016178342	0.019108504	0.030925544	0.04367867	0.018788606	0.040473047
CD14+CD16+	0.019536211	5.40E-10	0.026641701	0.0003269	0.00260488	0.028074801	0.000168061	0.005911568	0.010408015
CD14-CD16+	0.001489701	3.84E-09	0.088440342	1.69E-05	1.45E-08	1.44E-05	3.74E-09	0.000821649	0.01134787

Table S5: **Most significant p-values for total N_e and distinct immune cell populations during stage 2**

Cell Population	N01	N02	N03	N04	N05	N09	N10	N12	Average p-val
B	1.33E-10	4.15E-06	5.33E-06	6.30E-06	6.18E-09	3.23E-05	0.00050453	1.81E-08	6.91E-05
NK	3.12E-07	1.55E-15	0.0015218	8.83E-06	0	6.59E-05	3.67E-05	0.000417509	2.56E-04
TotalCD4	0.000143575	1.15E-13	1.45E-07	2.49E-09	1.21E-10	7.92E-07	0	0.001018788	1.45E-04
NaiveCD4	7.90E-05	8.10E-13	2.25E-08	1.37E-08	1.80E-10	5.00E-08	1.33E-15	0.000715953	9.94E-05
CMCD4	0.000501238	0	1.63E-05	5.66E-06	2.23E-10	1.58E-09	1.07E-11	0.015468996	2.00E-03
EMCD4	2.39E-05	6.85E-05	6.19E-07	1.53E-05	0.003591615	5.17E-05	4.28E-05	1.24E-05	4.76E-04
TotalCD8	4.14E-05	0	4.38E-06	2.01E-08	2.47E-08	3.28E-07	1.81E-07	0.000122706	2.11E-05
NaiveCD8	0.000276772	1.69E-14	0.000243409	3.12E-06	0.000170244	7.00E-06	2.25E-05	0.001554127	2.85E-04
CMCD8	0.000231924	0	0.000219593	1.41E-09	1.31E-05	4.20E-12	1.24E-08	1.69E-05	6.02E-05
EMCD8	2.61E-06	7.77E-15	7.98E-05	5.17E-06	2.88E-10	1.84E-05	2.21E-06	1.57E-06	1.37E-05
TotalMonos	0.001198301	0.049541077	0.000964758	7.69E-06	0.011460209	3.27E-10	5.26E-06	0.00012239	7.91E-03
CD14+CD16-	0.001874837	6.05E-06	0.004874093	0.000188553	7.12E-05	2.10E-09	2.78E-06	5.35E-08	8.77E-04
CD14+CD16+	0.001300999	0.000233735	0.002043388	5.49E-08	7.35E-08	0.000117594	0.000137797	1.00E-05	4.80E-04
CD14-CD16+	0.020717414	2.59E-07	6.41E-06	1.69E-06	3.71E-05	2.81E-09	5.75E-05	0.033168254	6.75E-03

Table S6: **Maximal Wavelet Cross-Spectrum (WCS) Amplitude averaged in time and across all macaques (see Methods)**

Cell population	Total	Plasma	BALF	BM	CD3	CD14
B	8.13E+05	1.41E+05	9.97E+04	4.51E+04	6.26E+04	4.61E+04
NK	6.48E+05	1.10E+05	9.29E+04	4.35E+04	5.86E+04	3.70E+04
Total CD4	9.31E+05	1.79E+05	1.27E+05	7.37E+04	8.60E+04	5.47E+04
Naive CD4	7.23E+05	1.39E+05	9.95E+04	5.75E+04	6.67E+04	4.22E+04
CM CD4	2.31E+05	4.20E+04	3.02E+04	1.60E+04	2.21E+04	1.33E+04
EM CD4	1.25E+04	3.43E+03	2.25E+03	1.44E+03	1.36E+03	8.93E+02
Total CD8	1.60E+06	2.61E+05	2.25E+05	8.54E+04	1.36E+05	7.11E+04
Naive CD8	6.91E+05	1.02E+05	8.56E+04	3.92E+04	6.19E+04	2.64E+04
CM CD8	4.25E+05	7.04E+04	7.17E+04	2.01E+04	3.44E+04	2.07E+04
EM CD8	8.48E+05	1.38E+05	9.56E+04	4.32E+04	7.62E+04	3.51E+04
Total Monos	1.56E+06	2.47E+05	2.52E+05	9.53E+04	1.50E+05	9.04E+04
CD14+CD16-	1.16E+06	1.90E+05	1.84E+05	7.62E+04	1.14E+05	7.07E+04
CD14+CD16+	3.42E+05	5.20E+04	5.25E+04	1.81E+04	3.08E+04	1.51E+04
CD14-CD16-	3.02E+05	4.19E+04	4.68E+04	1.43E+04	2.28E+04	1.20E+04
Tiss Avg	6.86E+05	1.14E+05	9.77E+04	4.19E+04	6.16E+04	3.57E+04

Table S7: **Model variables and parameters**

Variable/Parameter	Meaning	Value
$X(t)$	CD4+ T-cells	–
$Y_i(t)$	Virus strain i, $i = 1, \ldots, 2^{n+k}$ (infected cells)	–
$Z_j(t)$	CD8+ T-cells for epitope j, $j = 1, \ldots, n$	–
$W(t)^3$	Innate immune response	–
b	CD4 recruitment rate	5×10^4 [2]
c	CD4 decay rate	0.01 [1]
${\beta_0}^1$	wild-type viral replication rate	1.8×10^{-5} [2] [2]
δ_i	viral/infected cell decay rate	0.5 [10]
q_j	CD8 activation conversion	1.5,
r_j	CD8 epitope interaction rate	$0.5 \times 10^{-4} \leq r_j \leq 1.05 \times 10^{-4}$
μ_j	CD8 decay rate	0.02 [1]
q	Innate activation conversion	1.5
r	Innate-virus interaction rate	1.1×10^{-3}
μ	Innate decay rate	2
${h_0}^3$	pyroptosis of CD4-cells	0.01
${c_0}^3$	help by CD4-cells	5×10^{-7}
κ^1	fitness cost of each epitope	0.05
ϵ	base mutation probability rate	1.67×10^{-4} [7]
n, k	# of epitopes, neutral loci	$4 \leq n, k \leq 7$

[1] Note that $\beta_i = (1-\kappa)^d \beta_0$, where d is number of epitope mutations, in example simulations of full model. In simulations of base model (Figure S10), we consider more general pairwise random epistatic fitness landscape, $\beta_i = \left[\sum_{i_j=1}(1-\kappa_j) + \sum_{i_j=1, i_k=1} A_{jk}\right] \beta_0$, where additive fitness costs κ were uniformly distributed and pairwise interaction A_{jk} is normally distributed.

[2] Viral replication rate $\beta_0 = \frac{p}{\gamma} k_0$, where $V(t) = \frac{p}{\gamma} Y(t)$ is rescaling with V = viral load, $p = 2300$ viral production rate, $\gamma = 23$ virion decay rate, k_0 = virus infection rate.

[3] In base model, these quantities are taken zero.

Table S8: **Pathology of macaques N01 and N03 at time of necropsy**

Macaque	Diagnosis or outcome	Symptom(s) or gross pathology	Age (yr)	
			At time of infection	At necropsy
N01	SAIDS	Severe granulomatous lymphadenitis, thymitis, osteomyelitis, entercolitis, hepatitis, nephritis, and lymphangitis<br>Moderate ganglionitis<br>Severe Mycobacterium avium infection	6.6	7.9
N03	SAIDS	Severe enteritis, typhlocolitis, and splenitis<br>Moderate hepatitis and splenitis<br>Mild lymphoplasmacytic myocarditis and interstitial pneumonia	4.5	6.0

Table S9: **Phylogenetic and temporal resolution of SIV sequences from individual tissues and cell populations.**

**Macaque**	**Tissue or Cell Population**	**Number of Taxa**	**Likelihood Mapping[1]**	**Linear Regression[2]**	
			**% Unresolved Taxa**	**Slope**	**R^2**
	BAL	104	8.8	6.42E-05	0.61
	Bone Marrow	86	9.0	4.87E-05	0.22
N01	CD14	32	11.1	8.35E-05	0.4
	CD3	89	8.5	6.54E-05	0.35
	Plasma	185	4.0	8.62E-05	0.26
	BAL	77	11.0	6.33E-05	0.19
	Bone Marrow	75	7.5	2.10E-04	0.6
N02	CD14	63	6.5	1.84E-04	0.55
	CD3	74	10.4	9.30E-05	3.80E-01
	Plasma	120	6.6	2.58E-04	0.57
	BAL	89	8.9	9.82E-05	0.55
	Bone Marrow	95	7.8	6.91E-05	0.43
N03	CD14	41	10.2	7.46E-05	0.47
	CD3	97	8.2	8.11E-05	0.52
	Plasma	125	7.3	7.10E-05	0.3
	BAL	72	8.4	1.12E-04	0.34
	Bone Marrow	63	8.1	8.45E-05	0.15
N04	CD14	58	8.7	1.45E-04	0.56
	CD3	70	9.3	1.55E-04	0.51
	Plasma	94	7.5	1.40E-04	0.6
	BAL	80	9.9	4.57E-05	7.80E-02
	Bone Marrow	86	10.7	1.00E-04	0.47
N05	CD14	29	9.5	1.64E-04	0.55
	CD3	79	11.0	9.07E-05	4.80E-01
	Plasma	98	10.4	1.57E-04	0.54
	BAL	70	11.3	6.28E-05	0.18
	Bone Marrow	90	8.8	8.35E-05	0.29
N09	CD14	91	8.4	1.37E-04	0.33
	CD3	88	8.4	7.60E-05	9.40E-02
	Plasma	90	8.1	1.93E-04	0.77
	BAL	58	13.2	1.30E-04	0.18
	Bone Marrow	81	12.2	1.42E-04	0.38
N10	CD14	44	12.2	1.04E-04	0.51
	CD3	84	8.9	1.33E-04	0.28
	Plasma	101	10.4	1.48E-04	0.44
	BAL	71	14.7	1.52E-04	0.57
	Bone Marrow	81	16.8	2.76E-04	0.65
N12	CD14	59	14.3	1.47E-04	0.58
	CD3	82	12.4	2.09E-04	5.90E-01
	Plasma	96	12.0	1.96E-04	0.61

[1] The proportion of taxa for which an evolutionary relationship could not be resolved confidently was determined using likelihood mapping [15] in IQ-TREE v1.5.3 [9].

[2] The slope and coefficient of determination (R^2) of the linear regression model of increasing divergence over time from the most recent common ancestor (SIVmac251 viral swarm) were determined using the heuristic residual mean squared error estimator in TempEst [13].

Table S10: **GenBank accession numbers for viral sequence data.**

Origin	Accession
VS	KR999728 - KR999900
N01	KR999138 - KR999327
N02	KR999328 - KR999551
N03	MG931034 - MG931480
N04	KX068503 - KX068591; KX081437 - KX081454; KX081524 - KX081576; KX081781 - KX081799; KX081884 - KX081956; KX082195 - KX082210; KX082272 - KX082364
N05	KX068592 - KX081229; KX081455 - KX081478; KX081577 - KX081618; KX081800 - KX081839; KX081957 - KX082028; KX082211 - KX082228; KX082532 - KX082629
N09	KX081254 - KX081353; KX081479 - KX081498; KX081619 - KX081702; KX081840 - KX081862; KX082029 - KX082107; KX082229 - KX082254; KX082428 - KX082531
N10	KR999552 - KR999727
N12	KX068486 - KX068502; KX081354 - KX081436; KX081499 - KX081523; KX081703 - KX081780; KX081863 - KX081883; KX082108 - KX082194; KX082255 - KX082271; KX082365 - KX082427

References

[1] Christian L Althaus and Rob J De Boer. Dynamics of immune escape during hiv/siv infection. *PLoS computational biology*, 4(7):e1000103, 2008.

[2] Christian L Althaus and Rob J De Boer. Implications of ctl-mediated killing of hiv-infected cells during the non-productive stage of infection. *PLoS One*, 6(2):e16468, 2011.

[3] H.W. Van Deutekom, G. Wijnker, and R.J. De Boer. The rate of immune escape vanishes when multiple immune responses control an hiv infection. *Journal of immunology*, 191:3277–3286, 2013.

[4] A. J. Drummond and A. Rambaut. Beast: Bayesian evolutionary analysis by sampling trees. *BMC Evol Biol*, 7:214, 2007. ISSN 1471-2148 (Electronic) 1471-2148 (Linking). doi: 10.1186/1471-2148-7-214. URL https://www.ncbi.nlm.nih.gov/pubmed/17996036.

[5] A. J. Drummond, M. A. Suchard, D. Xie, and A. Rambaut. Bayesian phylogenetics with beauti and the beast 1.7. *Mol Biol Evol*, 29(8):1969–73, 2012. ISSN 1537-1719 (Electronic) 0737-4038 (Linking). doi: 10.1093/molbev/mss075. URL https://www.ncbi.nlm.nih.gov/pubmed/22367748.

[6] Stephen P Ellner and Lutz Becks. Rapid prey evolution and the dynamics of two-predator food webs. *Theoretical Ecology*, 4(2):133–152, 2011.

[7] Taylor A Kessinger, Alan S Perelson, and Richard A Neher. Inferring hiv escape rates from multi-locus genotype data. *Frontiers in immunology*, 4:252, 2013.

[8] P. Lemey, A. Rambaut, A. J. Drummond, and M. A. Suchard. Bayesian phylogeography finds its roots. *PLoS Comput Biol*, 5(9):e1000520, 2009. ISSN 1553-7358 (Electronic) 1553-734X (Linking). doi: 10.1371/journal.pcbi.1000520. URL https://www.ncbi.nlm.nih.gov/pubmed/19779555.

[9] L. T. Nguyen, H. A. Schmidt, A. von Haeseler, and B. Q. Minh. Iq-tree: a fast and effective stochastic algorithm for estimating maximum-likelihood phylogenies. *Mol Biol Evol*, 32(1):268–74, 2015. ISSN 1537-1719 (Electronic) 0737-4038 (Linking). doi: 10.1093/molbev/msu300. URL https://www.ncbi.nlm.nih.gov/pubmed/25371430.

[10] Cecilia Noecker, Krista Schaefer, Kelly Zaccheo, Yiding Yang, Judy Day, and Vitaly Ganusov. Simple mathematical models do not accurately predict early siv dynamics. *Viruses*, 7(3):1189–1217, 2015.

[11] Martin A Nowak, Robert M May, and Roy M Anderson. The evolutionary dynamics of hiv-1 quasispecies and the development of immunodeficiency disease. *Aids*, 4(11):1095–1104, 1990.

[12] Alan S Perelson, Avidan U Neumann, Martin Markowitz, John M Leonard, and David D Ho. Hiv-1 dynamics in vivo: virion clearance rate, infected cell life-span, and viral generation time. *Science*, 271 (5255):1582–1586, 1996.

[13] A. Rambaut, T. T. Lam, L. Max Carvalho, and O. G. Pybus. Exploring the temporal structure of heterochronous sequences using tempest (formerly path-o-gen). *Virus Evol*, 2(1):vew007, 2016. ISSN 2057-1577 (Linking). doi: 10.1093/ve/vew007. URL https://www.ncbi.nlm.nih.gov/pubmed/27774300.

[14] Hal L Smith and Patrick De Leenheer. Virus dynamics: a global analysis. *SIAM Journal on Applied Mathematics*, 63(4):1313–1327, 2003.

[15] K. Strimmer and A. von Haeseler. Likelihood-mapping: a simple method to visualize phylogenetic content of a sequence alignment. *Proc Natl Acad Sci U S A*, 94(13):6815–9, 1997. ISSN 0027-8424 (Print) 0027-8424 (Linking). URL https://www.ncbi.nlm.nih.gov/pubmed/9192648.

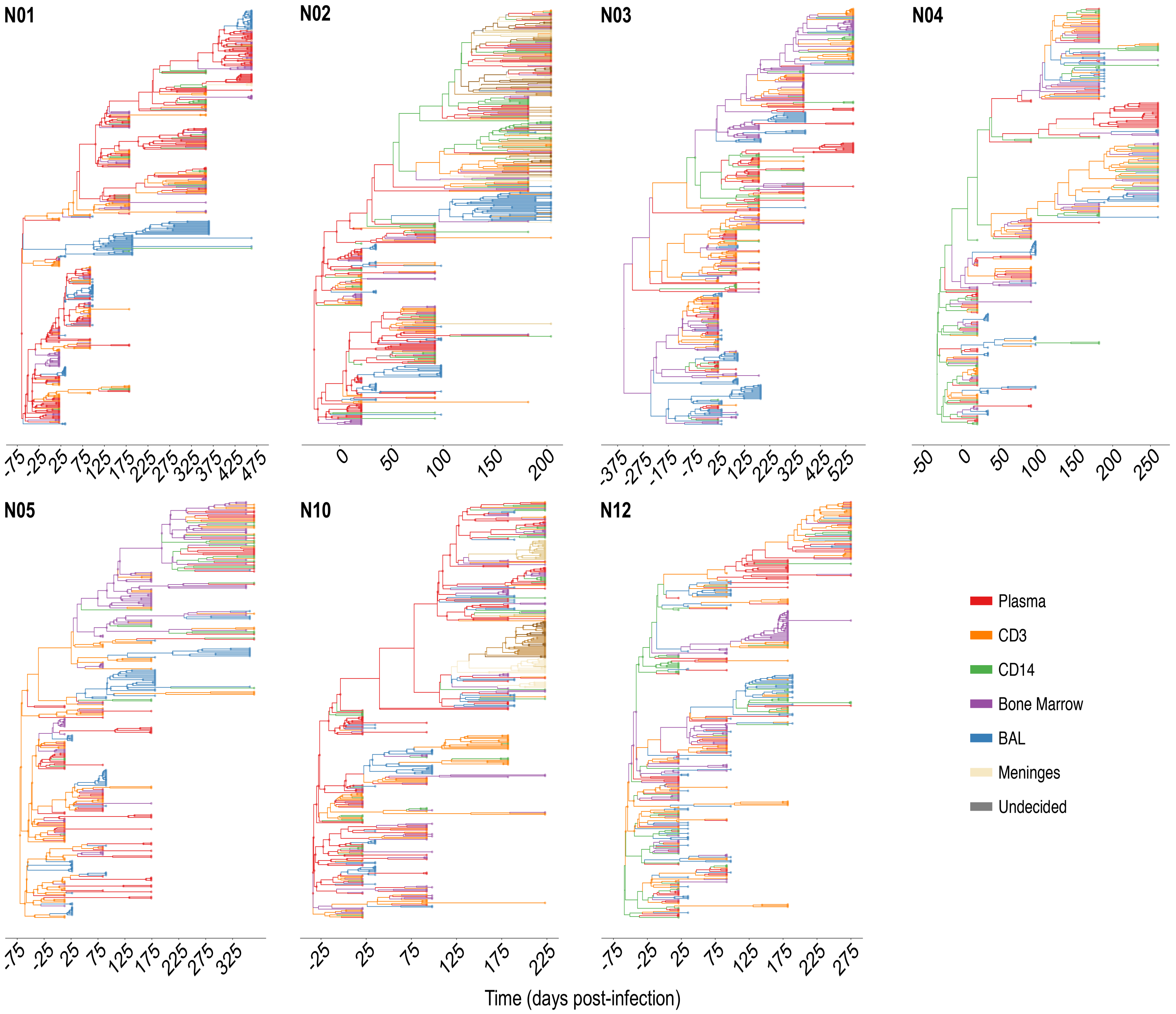

Figure S1: **Maximum clade credibility (MCC) trees for combined tissue locations from seven SIV-infected macaques.** *gp120* MCC trees were reconstructed using the Bayesian coalescent framework in BEAST v1.8 [4, 5] using all sampled tissues over time (x-axis). Branches are colored according to sampling origin (legend at right), with internal branches designated according to the highest posterior probability state using ancestral state reconstruction [8].

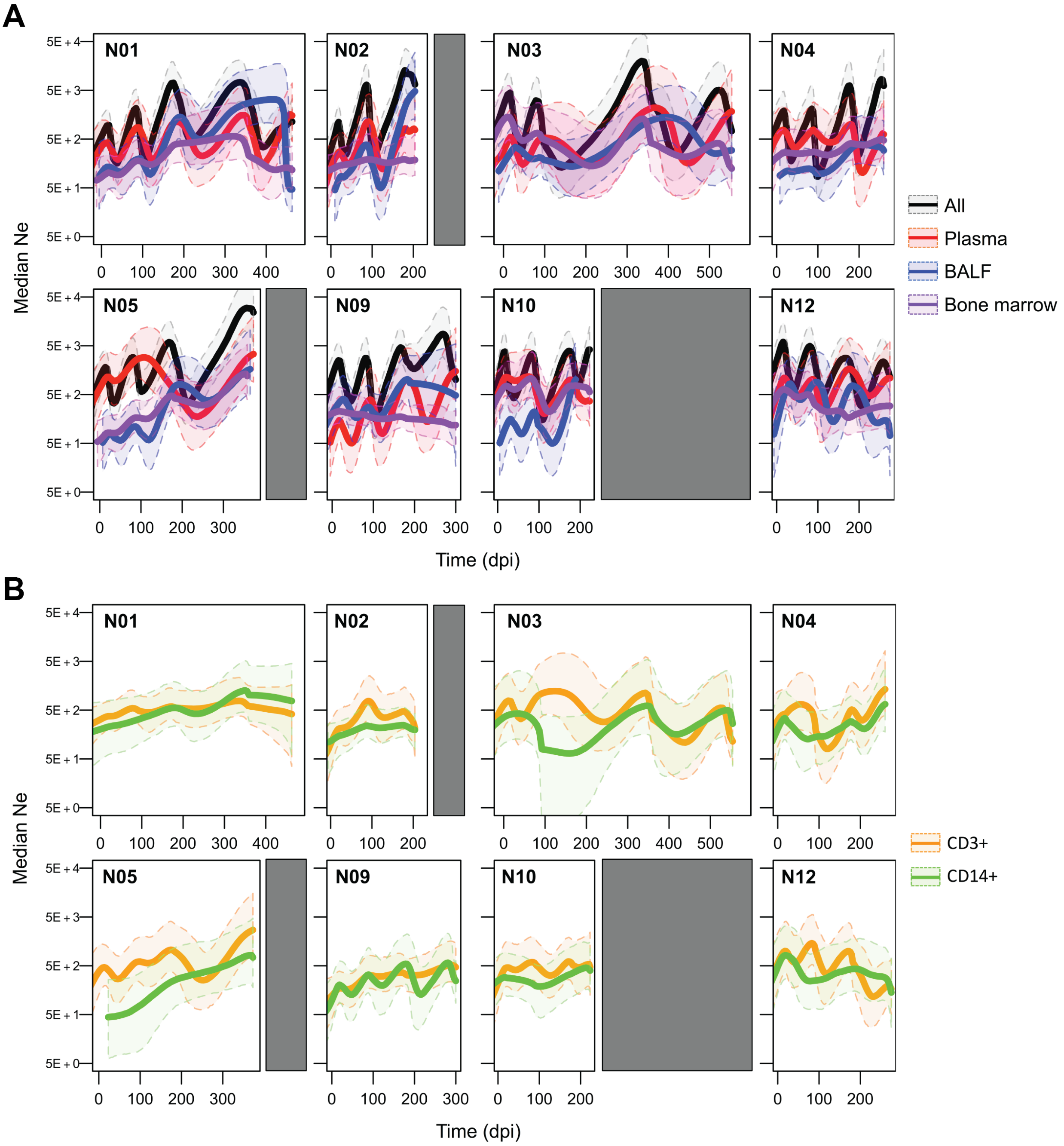

Figure S2: **Viral effective population sizes (N_e) for each macaque-specific set of longitudinally sampled locations.** Median N_e and high posterior density (HPD) intervals over time (x-axis) were inferred for all macaque *gp120* sequence alignments using the Bayesian coalescent framework in BEAST v1.8 [4, 5] using all ("All") and individually (colored accordingly) sampled tissue locations **(A)**. N_e for virus from sorted peripheral T cells and monocytes are depicted separately **(B)** to emphasize differences in cell populations from the same anatomical location - the peripheral blood.

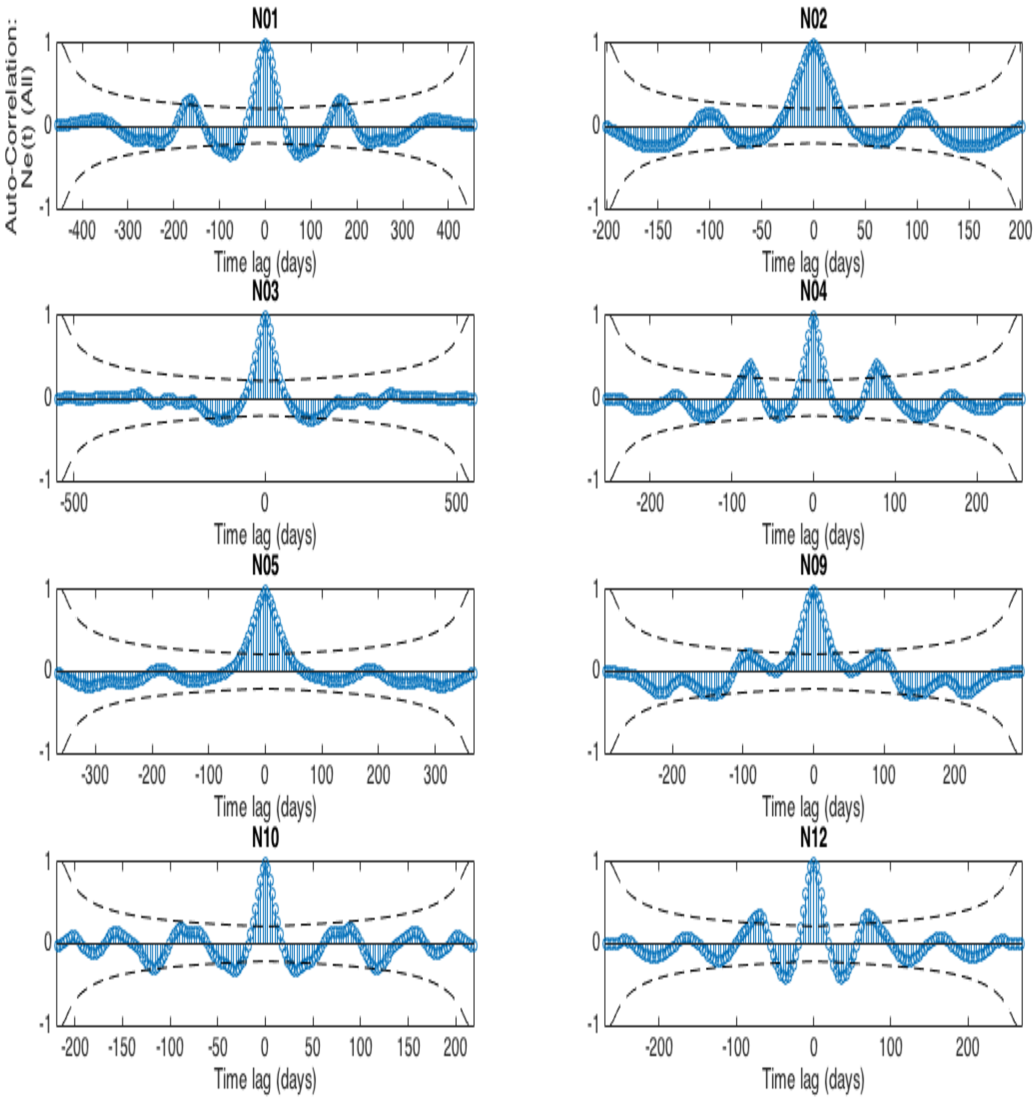

Figure S3: **Auto-correlation of total viral effective population size (N_e)**

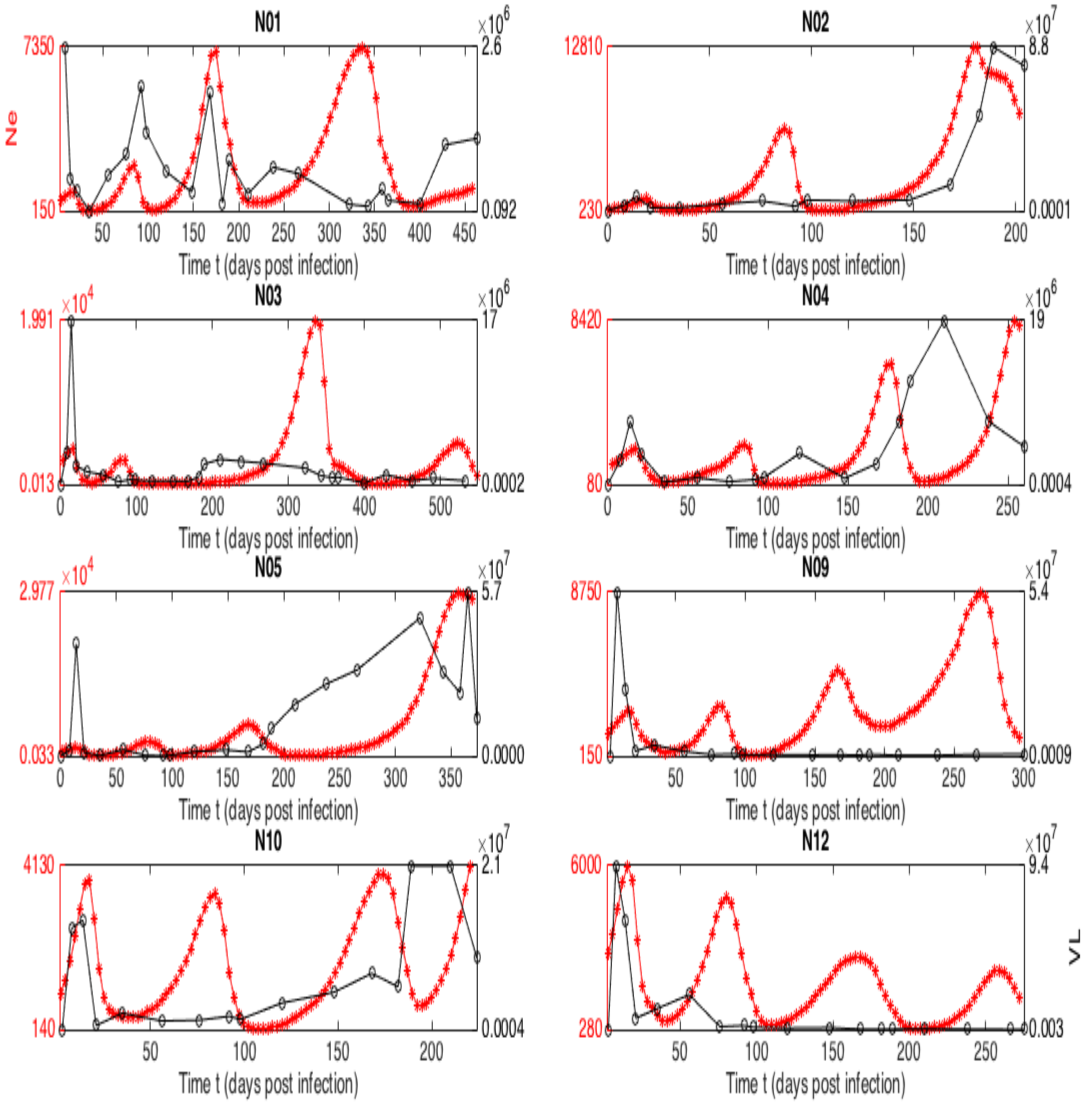

Figure S4: **Plot of Viral Load (VL) and combined N_e**

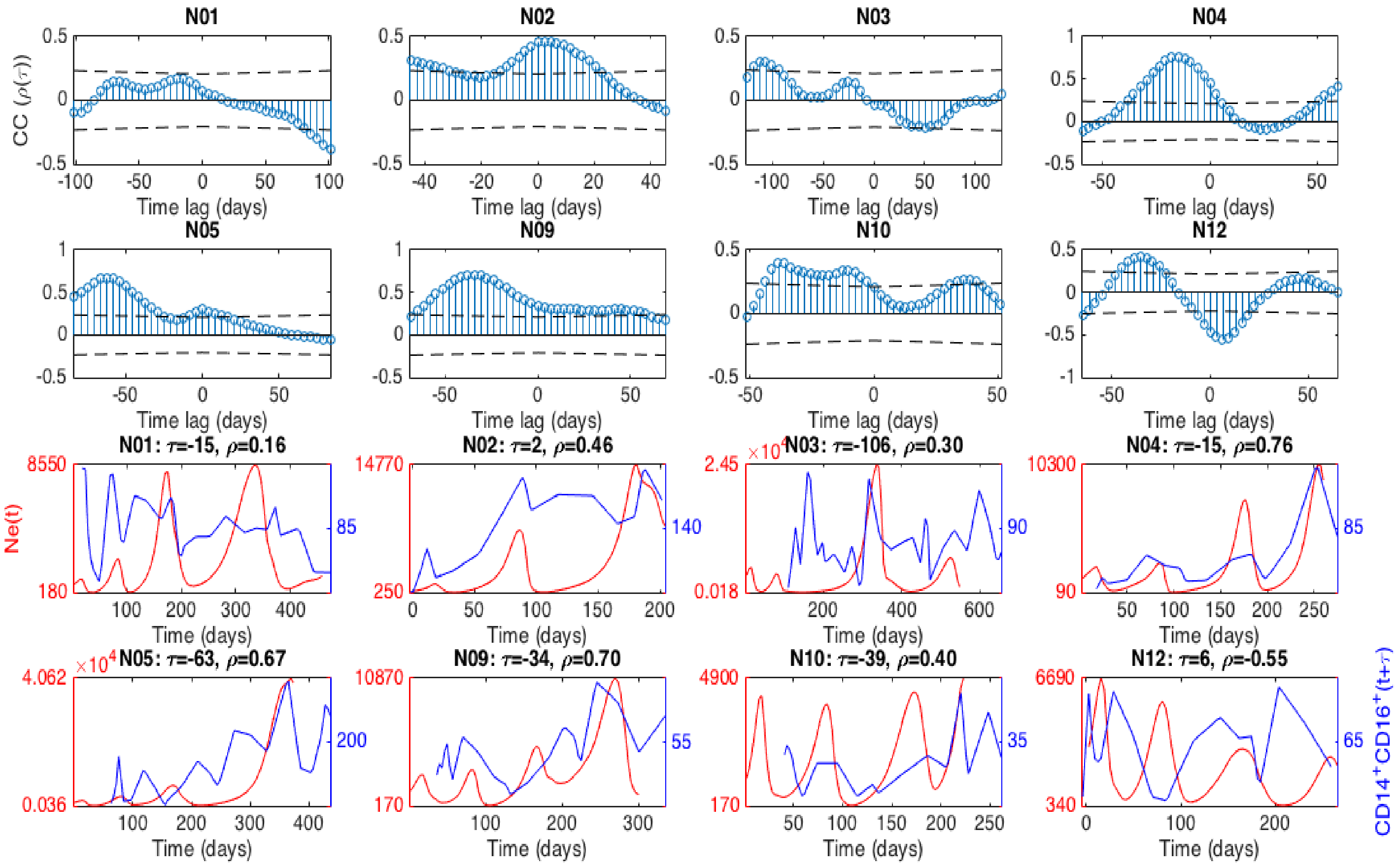

Figure S5: **Cross-correlation of combined N_e and CD14+16+ monocyte cells for each macaque (top), along with time-lagged plots (bottom) at most significant phase-shift.**

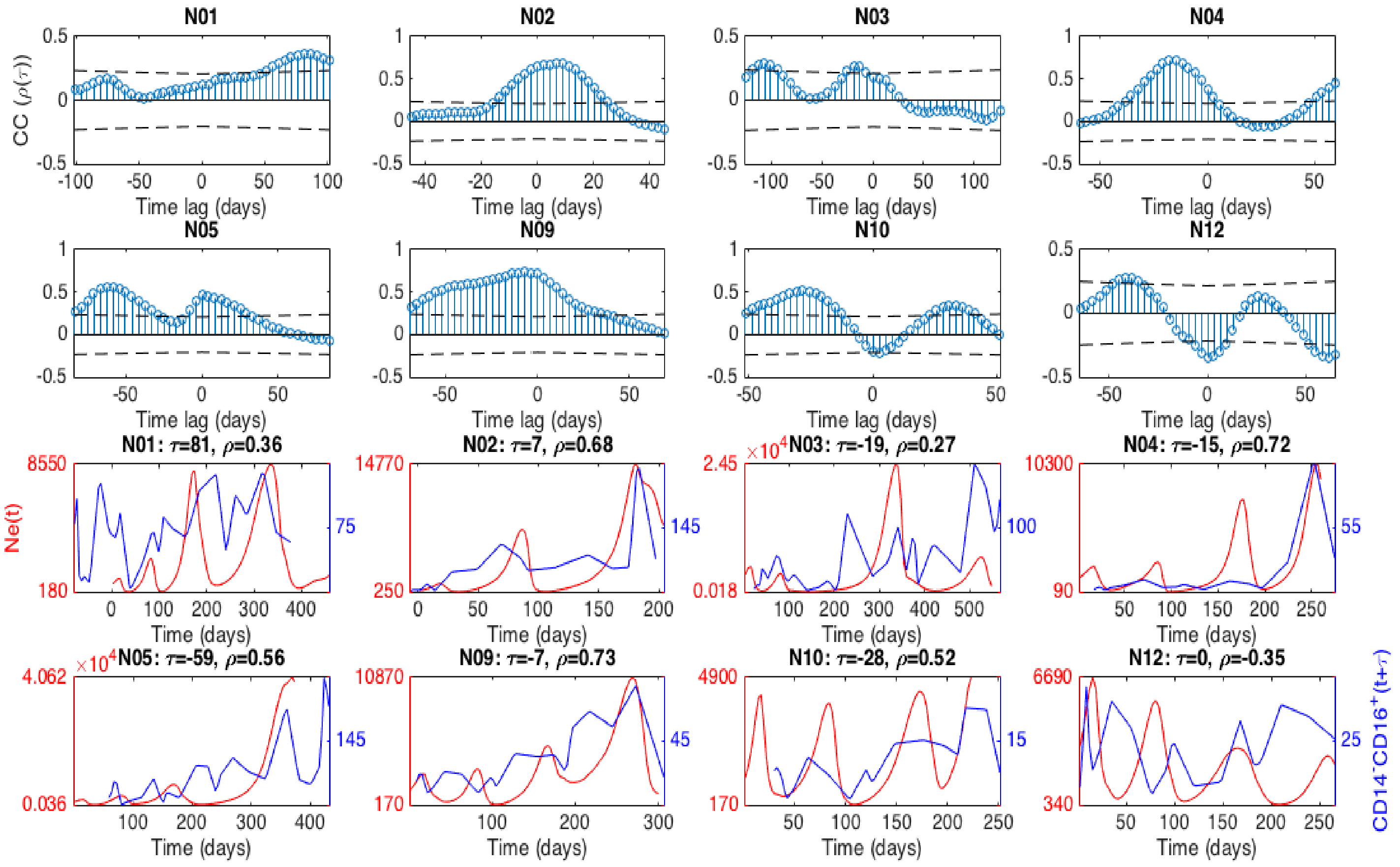

Figure S6: **Cross-correlation of combined N_e and CD14-16+ monocyte cells for each macaque (top), along with time-lagged plots (bottom) at most significant phase-shift.**

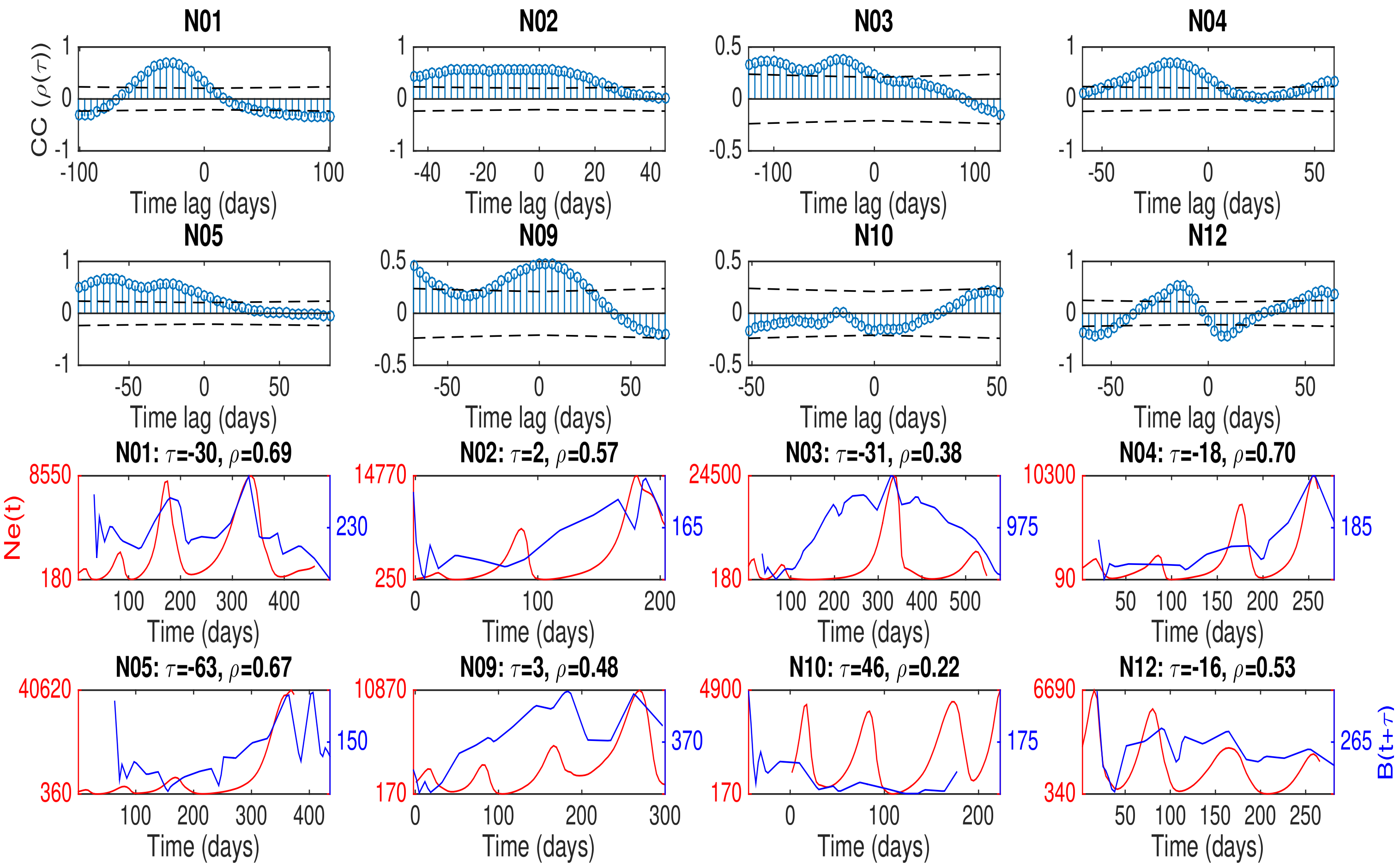

Figure S7: **Cross-correlation of combined N_e and B cells for each macaque (top), along with time-lagged plots (bottom) at most significant phase-shift.**

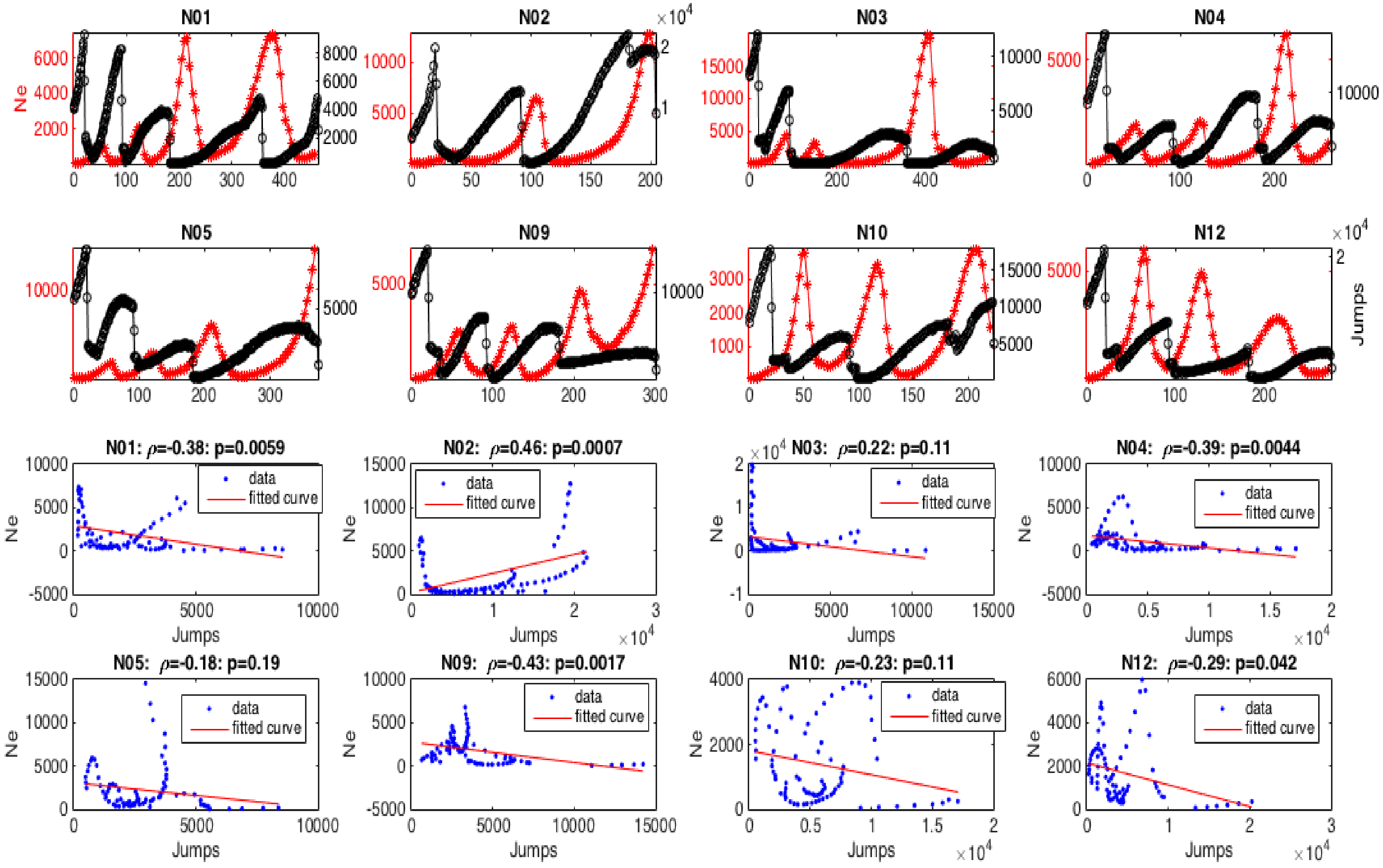

Figure S8: Plot of combined N_e and Markov jump data for each macaque (top), along with regression line and correlation (bottom).

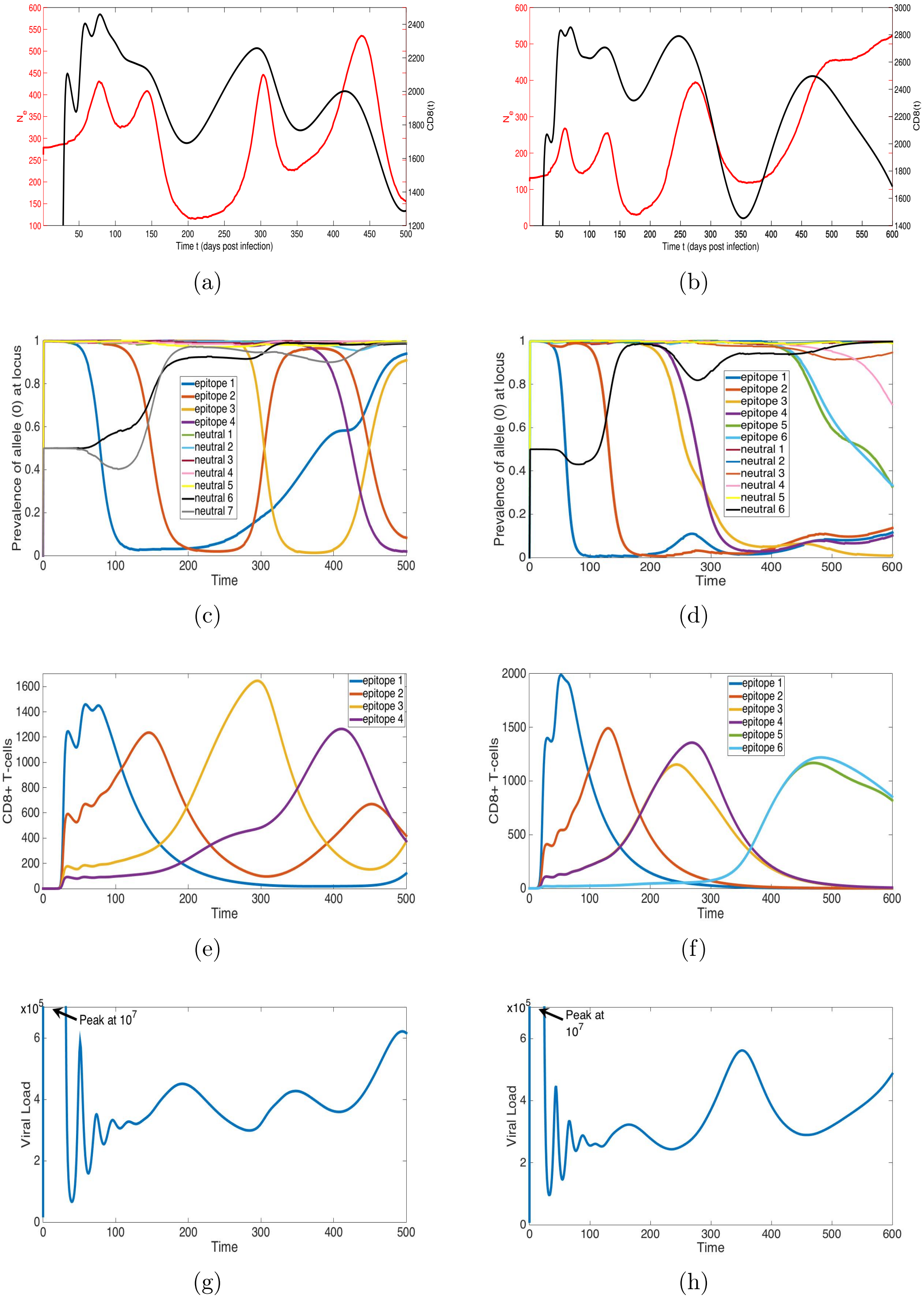

Figure S9: **Additional example simulations of full model.** (a),(b) Cross-correlation between N_e and total CD8+ T-cells; (c),(d) Prevalence of "0-allele" at each loci in viral (quasi-species) population; (e),(f) CD8+ T-cells responding to each epitope; (g),(h) Total viral load. (a),(c),(e),(g) are from example model simulation with $n = 4$ epitopes and immunodominance hierarchy given by $r_1 = 1.05, r_2 = 0.97, r_3 = 0.84, r_4 = 0.77$ $(\times 10^{-4})$, and (b),(d),(f),(h) are from example model simulation with $n = 6$ epitopes and immunodominance hierarchy given by $r_1 = 1.05, r_2 = 0.95, r_3 = r_4 = 0.84, r_5 = r_6 = 0.66$ $(\times 10^{-4})$

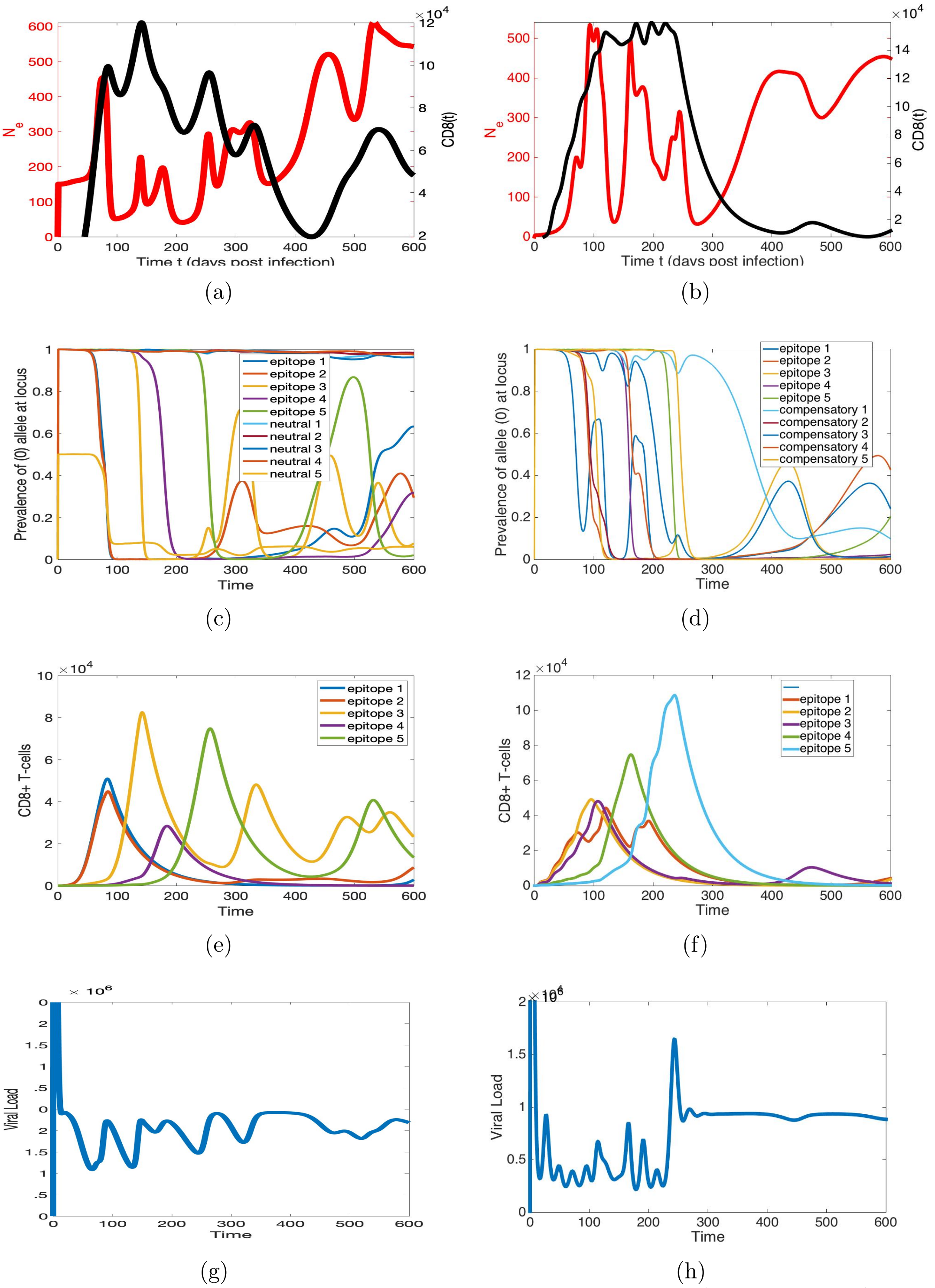

Figure S10: **Example model simulations of base model where $h_0 = 0$ (no pyroptosis), $c_0 = 0$ (no CD4 help) and $W = 0$ (no innate immune response), along with uniformly random distributed virus and immune fitness values, gaussian pairwise interactions between epitopes.** with $n = 5$ epitopes. (a),(b) Trajectories of N_e and total CD8+ T-cells for example model simulations; (c),(d) Prevalence of "0-allele" at each loci in viral (quasi-species) population; (e),(f) CD8+ T-cells responding to each epitope; (g),(h) Total viral load. (a),(c),(e),(g) are from example model simulation with $k = 5$ neutral allele, and (b),(d),(f),(h) are from example model simulation with $k = 5$ compensatory alleles which allow virus to gain back 95% of fitness cost. In simulations, additive viral fitness cost κ is uniformly distributed (as proportions of wild-type fitness) in the range 0 to 25% (1st panel) and 0 to 50% (2nd panel), with random additive pairwise epitope interactions that are normally distributed, along with immunodominance hierarchy (r_j) uniformly distributed in the range 0.5 to 1.05 $\times 10^{-4}$. Other non-zero parameters are same as in Table S6
